# The coordination chemistry and magnetism of some 3d–4f and 4f amino-polyalcohol compounds

**DOI:** 10.1016/j.ccr.2013.09.011

**Published:** 2014-02-01

**Authors:** Joseph W. Sharples, David Collison

**Affiliations:** School of Chemistry, The University of Manchester, Oxford Road, Manchester, Lancashire M13 9PL, United Kingdom

**Keywords:** tacn, 1,4,7-triazacyclononane, bmhH_2_, 1,2-bis(2-hydroxy-3-methoxybenzylidene)hydrazone, mshH, 3-methoxysalicylaldehyde hydrazone, 4-Me-py, 4-methyl-pyridine, chpH, 6-chloro-2-hydroxy-pyridine, acacH, acetylacetone, Δ*T*_*AD*_, adiabatic temperature change, a.c., alternating current, *U*_eff_, anisotropy barrier, *H*, applied magnetic field, *D*, axial anisotropy, HO_2_CPh, benzoic acid, Hbta, benzotriazole, *k*_*B*_, Boltzmann constant, BVS, bond valence sum, ^*n*^BudeaH_2_, n-butyldiethanolamine, CASPT2, complete active space perturbation theory 2, CASSCF, complete active space self consistent field, *J*_12_, coupling constant, d.c., direct current, *μ*_*β*_, electronic Bohr magneton, fcdcH_2_, ferrocene dicarboxylic acid, RdeaH_2_, functionalised diethanolamines, HDVV, Heisenberg Dirac van Vleck, ib, isobutyrate, ^i^PrO, isopropoxide, JT, Jahn–Teller, *g*, Landé *g*-value, −Δ*S*_*M*_, magnetic entropy change, MCE, magnetocaloric effect, mdeaH_2_, methyldiethanolamine, Micro-SQUID, micro-superconducting quantum interference device, *m*_*s*_, microstate, ^*n*^BudeaH_3_, *n*-butyldiethanolamine, edteH_4_, *N*,*N*,*N*′,*N*′-tetrakis(2-hydroxyethyl)ethylenediamine, sabheaH_3_, N-salicylidene-2-(bis(2-hydroxyethyl)amino)ethylamine, NMR, nuclear magnetic resonance, *χ″*, out-of-phase susceptibility, H_2_Pc, phthalocyanine, HO_2_C^*t*^Bu, pivalic acid, py, pyridine, QT, quantum tunnelling, RASSI, restricted active space state interaction, *E*, rhombic ZFS, SMM, single-molecule magnet, *χ*, susceptibility, *χT*, susceptibility temperature product, *Ŝ*_*n*_, spin operator, *τ*, tau, teaH_3_, triethanolamine, THF, tetrahydrofuran, tpaH, triphenyl acetic acid, ZFS, zero-field splitting, Lanthanide, 3d–4f, Triethanolamine, Diethanolamine, Coordination chemistry, Magnetism

## Abstract

Triethanolamine, teaH_3_, and diethanolamine, RdeaH_2_, 3d–4f and 4f compounds demonstrate an enormous variety in their structure and bonding. This review examines the synthetic strategies to these molecules and their magnetic properties, whilst trying to assess these ligands’ suitability towards new SMMs and magnetic refrigerants.

## Highlights

• teaH_3_ and RdeaH_2_ are popular ligands towards SMMs and magnetocaloric compounds.
• We review the synthesis routes to 3d−4f and 4f compounds with these ligands.
• We review the bonding of these ligands with metals in these compounds.
• We review the magnetic properties of these compounds as SMMs and refrigerants.
• We assess the suitability of these ligands to these applications.

## Graphical abstract

**Figure:**
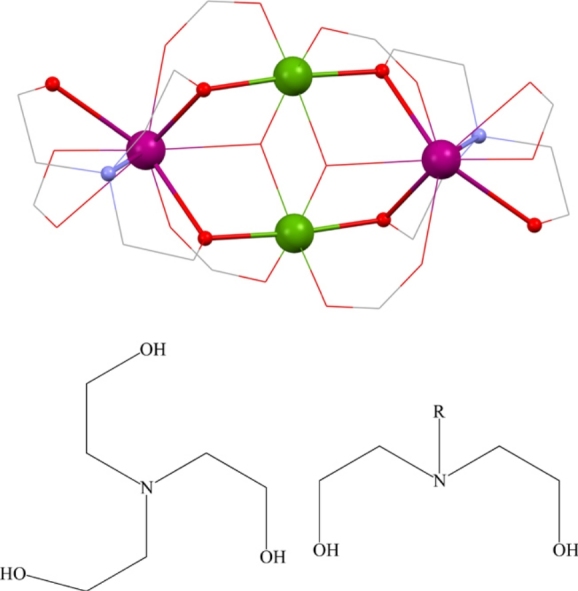

## 1 Introduction

### 1.1 Molecular magnetism and poly-alcohol ligands

Molecular magnetism is a wide ranging area of research that began around 20 years ago, primarily involving the synthesis and study of metal coordination compounds. Twin pillars of this effort, amongst others, are the discovery of single-molecule magnets (SMMs) [1], in which data can in principle be stored at a molecular level; and magnetic refrigerants [2], compounds with a large magnetocaloric effect (MCE) that can be used to cool to and below liquid ^4^He temperatures. Much early research in the former area involved transition metal compounds, such as [Mn^III^_8_Mn^IV^_4_O_12_(O_2_CCH_3_)_16_(H_2_O)_4_]·2CH_3_COOH·4H_2_O [3] and {[Fe^III^_8_O_2_(OH)_12_(tacn)_6_]Br_7_·H_2_O}Br·8H_2_O, where tacn is 1,4,7-triazacyclononane [4], whilst the latter topic involved cages such as [Fe^III^_14_O_6_(bta)_6_(OMe)_18_Cl_6_], where Hbta is benzotriazole [5]. Many subsequent efforts [*e.g.* 6] used polyalcohol pro-ligands, amongst others, to connect the metals into larger assemblies, so increasing the ground state spin, *S*, as this was initially believed to be the key to increasing the temperatures at which SMMs could maintain magnetised states to a practical level. The challenge of achieving this is still on-going. Selected examples were well reviewed by Brechin in 2005 [7], who showcased the SMM behaviour of mostly Mn^III^ and Fe^III^ cages. The first of these ions was particularly prevalent in early research, having a sizable single-ion anisotropy when in the octahedral configuration, which can define an Ising-type ground state. Introducing lanthanides into molecular magnetism, a more recent development, led to large improvements in the energy barriers of SMMs, on account of the anisotropy of Ln^III^ ions such as Dy^III^, Tb^III^ and Ho^III^, particularly by Ishikawa, with a [Tb^III^(Pc)_2_]^−^ compound [8]. The high spin and isotropy of Gd^III^ has achieved similarly impressive results in increasing the −Δ*S*_*M*_ (MCE) of molecule-based refrigerants, for example [Gd^III^_2_(O_2_CCH_3_)_6_(H_2_O)_4_]·4H_2_O [9].

Therefore, combined with the established benefits of appropriate d-transition metals, or as homometallic species, lanthanide-based compounds impart significant structural and magnetic properties, distinct from their 3d cousins, giving some of the best performing SMMs and magnetic refrigerants.

### 1.2 Amino-polyalcohol ligands

**Fig. 1:**
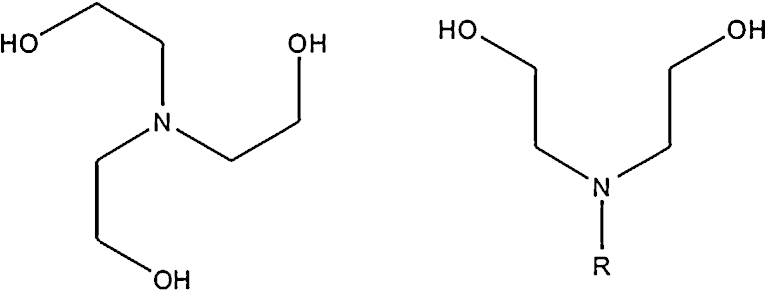
Left, teaH_3_, triethanolamine. Right RdeaH_2_, a generic functionalised diethanolamine.

An extension of this work uses amino-polyalcohol pro-ligands. These would seem an ideal continuation of previous efforts as they possess an affinity to lanthanides due to their O donors, but also incorporate a new N functionality which can take part in bonding, though to date there are significantly fewer 3d–4f and 4f compounds of this type than with 3d metals alone, so this is an ongoing area of research. The focus here will be on those compounds prepared with the pro-ligands teaH_3_, triethanolamine, and RdeaH_2_, functionalised diethanolamines, where R is H or C_*n*_H_2*n*+1_; these being shown in Fig. 1. These are flexible pro-ligands that can bond to metals in many ways, or with many “modes”. Their profligacy also stems from the way they exist in a variety of forms, depending on the basicity of the conditions, as singly, doubly or triply protonated species as appropriate, in addition to their completely deprotonated forms. Indeed, the teaH_3_ pro-ligand bonds to metals in each of the tea^3−^, teaH^2−^, teaH_2_^−^ and teaH_3_ forms in 3d metal chemistry in a wide variety of ways, also featuring as a teaH_4_^+^ cation [10]. Co-ordination occurs through O or OH arms and the N-donor, in addition to any other functionalities, with the flexible OH arms accommodating a large range of ionic radii of metals. These properties assist in a common technique in co-ordination chemistry, so-called “serendipitous” assembly [11], which involves combining reagents to form unpredictable products; beginning with flexible starting materials can improve one's chance of forming a product. Modifications of “successful” syntheses can then be used to explore further.

This review article examines the synthesis, structure and magnetic properties of 3d–4f and 4f coordination compounds with amino-polyalcoholate ligands and their magnetic properties. In addition to reviewing these we assess the suitability of these ligands to this research and why they are such a double-edged sword in the fight for improved SMM and MCE molecular materials.

## 2 Incomplete double cubanes

### 2.1 Beginnings: {Fe^III^_2_Ln^III^_2_}

**Fig. 2:**
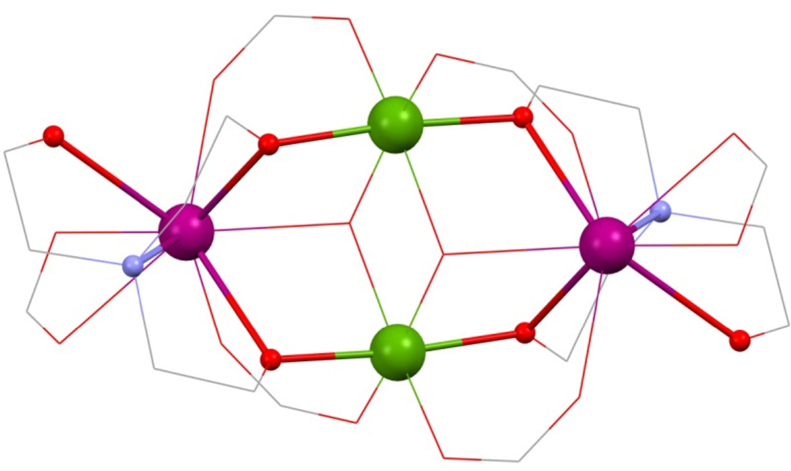
[Fe^III^_2_Ln^III^_2_(OH)_2_(teaH)_2_(O_2_CPh)_6_] **(2)**. Key: Ln^III^, purple spheres; Fe^III^, green; O, red (teaH^2−^) and wireframe (OH and benzoate); N, blue (teaH^2−^); C, grey wireframe; no H atoms, Ph or solvent groups are shown for clarity. See Ref. [12] for structural information.

Christou's group were the first to synthesise 3d–4f triethanolamine cages [12] in 2006, namely [Fe^III^_2_Ho^III^_2_(OH)_2_(teaH)_2_(O_2_CPh)_4_(NO_3_)_2_]·6MeCN **(1)** and [Fe^III^_2_Ln^III^_2_(OH)_2_(teaH)_2_(O_2_CPh)_6_]·4MeCN·3H_2_O **(2)** (Ln^III^ = Tb^III^ or Dy^III^ and HO_2_CPh is benzoic acid) giving the first Fe^III^-4f SMMs (see Fig. 2). All were prepared from a reaction of the iron triangle [Fe^III^_3_O(O_2_CPh)_6_(H_2_O)_3_](O_2_CPh), Ln^III^(NO_3_)_3_·*n*H_2_O, teaH_3_ and acetonitrile stirring at room temperature giving what is a common structural motif in 3d–4f teaH_3_ chemistry, and already known beyond this niche [13], [14]: the incomplete double-cubane. This is made up of two “M_4_O_4_” cubanes fused on one face and each missing a metal vertex, making a planar tetrametallic core. Alternative descriptions include a rhombus or butterfly. This structure, with several variations, is common to **(1)** and **(2)** and in more detail, for **(1)**, is thus: The planar core is composed of two “inner” octahedrally co-ordinated Fe^III^ ions which are formed into triangles by an OH group, bonding to two outer Ln^III^ ions with four carboxylate groups bonding between hetero-metals. These ligands “frame” the rhombus and lead to inter-molecular interactions as a result of the π–π stacking between aromatic groups.

**Scheme 1:**
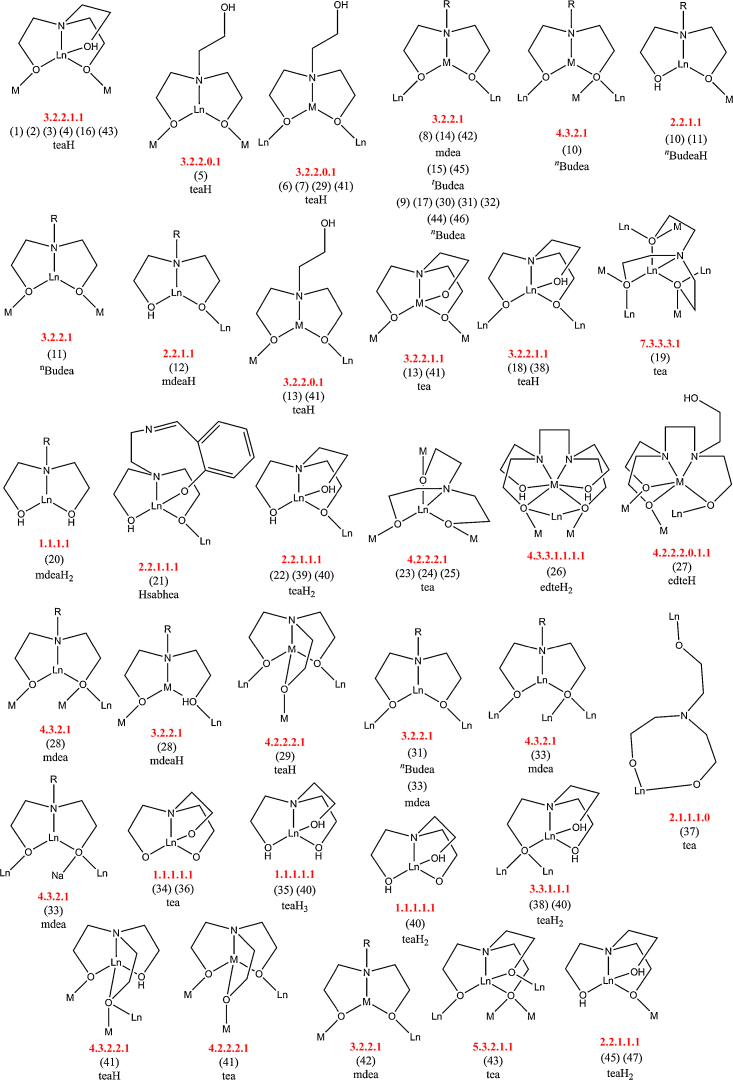
Bonding modes of the teaH_*x*_^(3−*x*)−^and RdeaH_*x*_^(2−*x*)−^ ligands in this review (numbered), and where M is a transition metal ion and Ln is a lanthanide; Harris notation describes the bonding of the ligands to these metals.

The singly protonated teaH^2−^ exhibits the bonding mode 3.2.2.1.1, described using Harris notation [15], used here throughout. This lists the total number of metals bonded, the number attached to each arm, then the number bonded to the N atom and is followed even when an arm is non-bonding, represented by a zero, and extended to other ligand types such as carboxylates and nitrates. Thus the aforementioned carboxylates show the 2.1.1 mode. Note that all modes of amine-polyol bonding in this review are collated in Scheme 1 for easy reference.

The teaH^2−^ ligand is centred on the outer lanthanide ions by the N-donor atom with two unprotonated arms linking this to each of the inner 3d metals. The third protonated arm bonds terminally to the same Ln^III^ ion. This is the most common bonding mode found where triethanolamine features in polymetallic compounds, often as part of much different topologies, *vide infra.* The H on the teaH^2−^ here is assigned by assuming that unprotonated arms bond to more metal ions, though only after metal oxidation states have been assigned by geometries (3d metals) or experience (Ln^III^). Filling the coordination sphere of the 4f ions is a nitrate (2.1.1.0), whereas in **(2)** these are replaced by capping 2.1.1 ^−^O_2_CPh ligands, the geometry around these ions being a capped square antiprism.

**Fig. 3:**
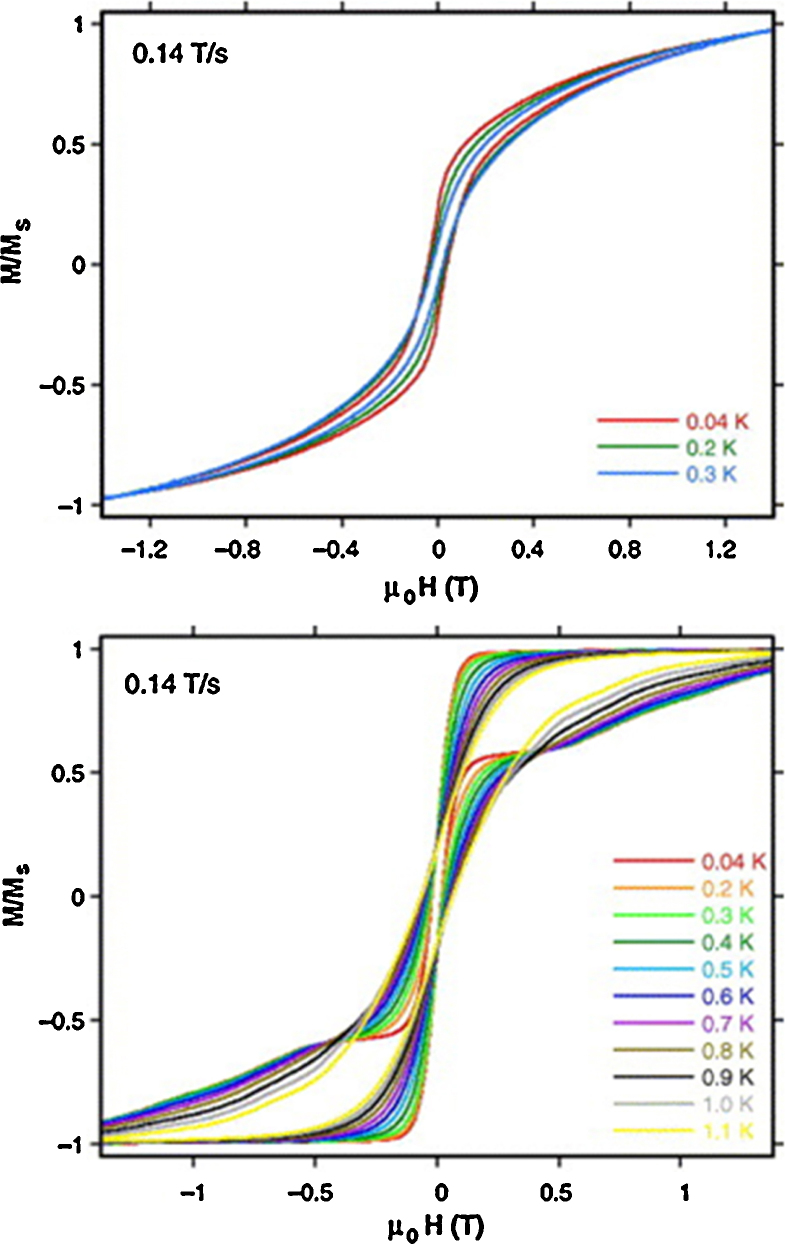
Upper panel, hysteresis loop for [Fe^III^_2_Ho^III^_2_(OH)_2_(teaH)_2_(O_2_CPh)_4_(NO_3_)_2_]·6MeCN **(1)** measured on single crystals at 0.04, 0.2 and 0.3 K for a sweep rate of 0.14 T s^−1^. Lower panel, the same for [Fe^III^_2_Dy^III^_2_(OH)_2_(teaH)_2_(O_2_CPh)_6_]·4MeCN·3H_2_O between 0.04 and 1.1 K **(Dy-2)**.

Adapted with permission from [12]. Copyright 2006, Elsevier.

Magnetic measurements show similar decreases in *χT* with decreasing temperature for **(1)** and **(Tb-2 and Dy-2)**, consistent with overall antiferromagnetic interactions between spins. *χT* falls gradually with decreasing temperature and then rapidly at low temperature, though this behaviour may also be assigned completely or in part to the anisotropy of the Ln^III^ ions and the depopulation of Stark levels, which precluded a fit of these data. a.c. susceptibility data hinted at SMM behaviour for **(1)** and **(Dy-2)**, with plots of *χ*″ *versus* temperature for different frequencies of applied field divergent at low temperatures, though no maxima were observed above 1.8 K. Further micro-SQUID measurements on **(1)** and **(Dy-2)**, though, revealed these compounds are SMMs by the discovery of hysteresis loops at lower temperatures. For the former, loops opening below 0.3 K were smooth (upper panel, Fig. 3) and assigned to the inter-molecular stacking interaction. Step-structured loops indicating a quantum tunnelling process (QTM) were found for the latter, opening below 1.1 K (lower panel, Fig. 3). The presence of this relaxation at zero-field gives a rapid decrease of the magnetisation and no *U*_eff_ values could be extracted.

Although the isotropic Gd^III^ analogues were not prepared, which could be used to assess the extent of magnetic anisotropy involving the Ln^III^ ion, the likely origin is the 4f metal, notwithstanding the presence of anisotropy in some Fe^III^ cages, which is dependent on geometry and factors of molecular symmetry. The rapid quantum tunnelling, though, is a feature noted elsewhere in Ln^III^ SMMs [*e.g.* 16].

### 2.2 Variations on a theme: {Mn^III^_2_Gd^III^_2_}

Near-identical analogues of **(1)** and **(2)** were found with different d-transition metals, such as [Mn^III^_2_Gd^III^_2_(OH)_2_(teaH)_2_(O_2_CPh)_4_(NO_3_)_2_] **(3)** [17], also from Christou's group, and part of a series involving other polyols. Only the one lanthanide compound was reported, prepared from a modification of the above reaction by using [Mn^II^Mn^III^_2_O(O_2_CPh)_6_(py)_2_(H_2_O)], where py is pyridine. The structure is the same as **(1)** with the presence of H on the teaH^2−^ ligand assigned after confirmation of the Mn^III^ oxidation state by Bond Valence Sum (BVS) analysis and geometries. These ions also show characteristic Jahn–Teller distortion down the O(O_2_CPh)—Mn^III^—OH axis. H-bonds between the OH of the teaH^2−^ arm and the oxygen atom (HO⋯H) of a nitrate group form a supra-molecular chain structure.

Qualitatively similar behaviour in *χT*(*T*) to **(1)** and **(2)** was assigned to the intra-molecular H-bonding interaction, though fits of these data could not be obtained using a HDVV spin Hamiltonian, which was reported to be due to the presence of weak interactions between spins, which would help to quantify exchange interactions and any anisotropy present. a.c. susceptibility data suggested an ill-defined *S* = 4 ground state from the frustrated triangular Mn^III^_2_Gd^III^ topology, though no SMM behaviour was discovered. The lack of SMM behaviour here suggests the importance of the anisotropic Ln^III^ ions and so most likely the slow relaxation in **(1)** and **(2)** is due to the 4f ions.

### 2.3 Mössbauer and EPR analysis of {Fe^III^_2_Ln^III^_2_}

Powell's group investigated the effects of the *para*-R substituent in [Fe^III^_2_Dy^III^_2_(OH)_2_(teaH)_2_(O_2_CPhR)_6_] **(4)** [18] (R = H, Me, ^*t*^Bu, NO_2_ or CN) on the coupling between the 3d and 4f ions. This variation in R does nothing to change the overall molecular structure, which is identical to those seen in **(2)**, and prepared in a similar way, with slight variations in solvent. Using ^57^Fe Mössbauer spectroscopy a significant change in the internal field at the ^57^Fe ion was detected, and ascribed to the differing environment of the Dy^III^ ions, the ^57^Fe “electron cloud” being polarised by their influence.

**Fig. 4:**
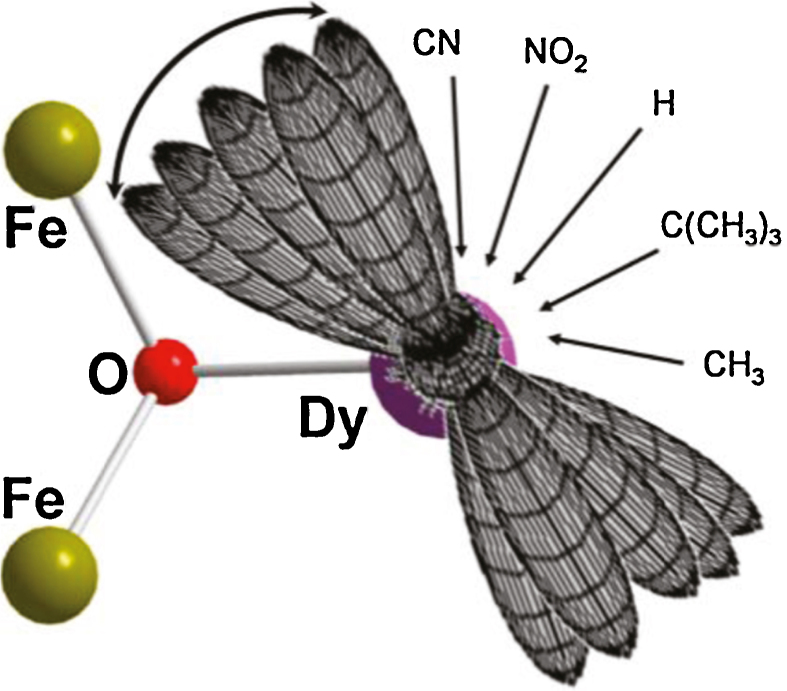
Orientation of the anisotropy axes of Dy^III^ in [Fe^III^_2_Dy^III^_2_(OH)_2_(teaH)_2_(O_2_CPhR)_6_] **(4)** for the given R groups, CN, NO_2_, H, ^*t*^Bu and CH_3_, from left to right. Key: atoms as labelled.

Reprinted with permission from [18]. Copyright (2011) American Chemical Society.

The Fe^III^ spins cancel in an Y^III^ analogue **(Y-4)**, giving an *S* = 0 ground state, so allowing the results of the changing orientation of the Dy^III^'s principal magnetic anisotropy axes to be probed (shown in Fig. 4 for each substituent). Thus the influence of a ligand substituent on the Dy^III^ ion can have a significant effect on the magnetic properties overall. The ultimate application of this Mössbauer technique is the determination of the orientation of the magnetisation axes on anisotropic ions.

The syntheses of **(1)–(4)** have relied on beginning with a 3d-metal triangle, which appears to act as a scaffold for incorporating lanthanides around a part of it, after breaking up in solution, at room temperature, then capping the high coordination number 4f metal with the bulky co-ligands added. This is different from the following **(5)**, where the components are added separately to achieve a different result.

Briefly, recent work, explained in much greater detail in [19] on the compounds [Fe^III^_2_Ln^III^_2_(OH)_2_(teaH)_2_(O_2_CPh)_6_], where Ln^III^ is Ce^III^—Yb^III^ (excluding Pm^III^) or Y^III^, is also particularly interesting. This utilised EPR (Electron Paramagnetic Resonance spectroscopy) on the latter **(Y-4)**, amongst other investigations with variable temperature X-band data used to determine *D*, *E* and *g* values for the Fe^III^ ions. With **(Nd-4)** and **(Dy-4)**, this revealed the importance of Ln^III^—Ln^III^ dipole–dipole interactions at less than 20 K.

### 2.4 Inside-out: {Co^III^_2_Ln^III^_2_}

Further variations with 3d-transition metals are possible: from the Murray group, stirring Co^II^(NO_3_)_2_·6H_2_O, Ln^III^(NO_3_)_3_·*n*H_2_O, teaH_3_, benzoic acid and the base triethylamine in acetonitrile at room temperature gave the double cluster compound [Co^III^_2_Ln^III^_2_(OMe)_2_(teaH)_2_(O_2_CPh)_4_(MeOH)_4_](NO_3_)_2_·MeOH·H_2_O:[Co^III^_2_Ln^III^_2_(OMe)_2_(teaH)_2_(O_2_CPh)_4_(MeOH)_2_(NO_3_)_2_]·MeOH·H_2_O **(5)** [20] (Ln^III^ is Gd^III^, Tb^III^ or Dy^III^) where Co^II^ is oxidised in air to Co^III^ during the reaction. Significant differences were found compared with **(1–4)**, though **(5)** too has an incomplete double cubane core. Notably, two forms exist in the crystal structure in a 1:1 ratio: a further difference is that here we find the Ln^III^ ions as the central pair and the Co^III^ ions as the outer metals, OMe groups bridging a Ln^III^_2_Co^III^ triangular arrangement. The teaH^2−^ ligand here has the bonding mode 3.2.2.0.1 and is centred by the N-donor atom on Co^III^ so it links the outer and inner metals as before, but the protonated arm is unbound. Most likely this arm is unable to add to the already complete co-ordination sphere of a 3d metal, unlike when centred on Ln^III^, with its larger ionic radius and coordination number. In one of the structural pair, two methanol groups bond to each Ln^III^, but in the second compound one is replaced by a nitrate, with resulting nitrate:methanol H-bonding. Though these lanthanide(III) ions have qualitatively the same square-antiprismatic geometry, these are distorted away from regularity by differing amounts.

The reasoning behind the inversion in position of Co^III^ and Ln^III^ *cf.* Fe^III^ and Ln^III^ (*e.g.* **(1)**) is still unknown and not speculated upon by the authors, though there is an obvious dependence on synthetic route, judging by this and also the latest results from Powell [14]. This latter example is the only one so far of a controllable synthesis where the position of the 4f and 3d ions in incomplete double cubanes can be altered in their positions, though does not involve teaH_3_ or RdeaH_2_.

As this Co^III^ is diamagnetic the interpretation of the magnetic data is simplified to that of the central Ln^III^ dimer. For **(Gd-5)** the exchange constant was fitted using the isotropic Hamiltonian below (Eq. (1)), incorporating a Zeeman term, where *J*_12_ is the exchange between the inner 4f ions, ${\hat{S}}_{1}$ and ${\hat{S}}_{2}$ the spin operators for these ions, *g* the Landé *g*-value, *μ*_*β*_ the electronic Bohr magneton and *H* the applied magnetic field.

(1) $$\hat{H}=-2J_{12}{\hat{S}}_{1}{\hat{S}}_{2}+g\mu_{\beta }H({\hat{S}}_{1}+S_{2})$$

**Fig. 5:**
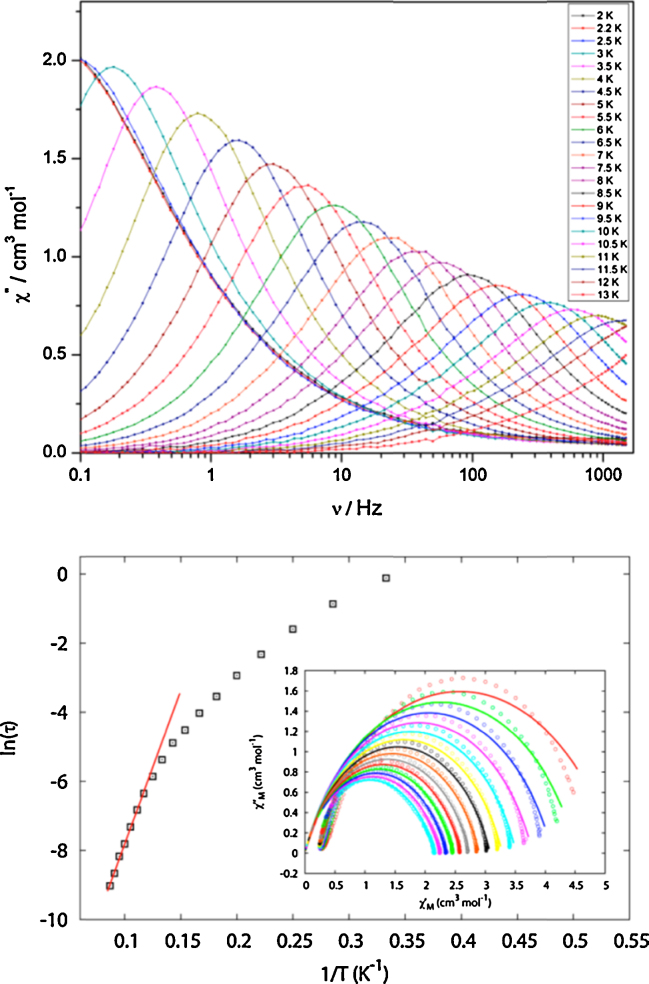
Upper panel, *χ*″ *versus ν*, frequency, for [Co^III^_2_Ln^III^_2_(OMe)_2_(teaH)_2_(O_2_CPh)_4_(MeOH)_4_](NO_3_)_2_·MeOH·H_2_O:[Co^III^_2_Ln^III^_2_(OMe)_2_(teaH)_2_(O_2_CPh)_4_(MeOH)_2_(NO_3_)_2_]·MeOH·H_2_O **(Dy-5)** between 2 and 13 K. Lower panel main, the Arrhenius plot and, inset, Cole-Cole plots between 4 and 10.5 K for the same compound. Solid lines are fits to the experimental results given in open spheres.

Reprinted with permission from [20]. Copyright (2012) American Chemical Society.

This yielded *J*_12_ = 0, *i.e.* the metals were non-interacting; hence the measured decrease in *χT* at low temperatures was assigned to depopulation of the *m*_*s*_ levels, rather than to antiferromagnetic coupling, though more significant exchange was found by fitting the results of *ab initio* calculations to magnetic data. For **(Tb-5)** and **(Dy-5)** similar depopulation, this time of the *m*_*J*_ states, was initially postulated; a.c. susceptibility data for the latter, **(Dy-5)**, showed clear frequency and temperature dependent *χ*″ maxima (upper panel, Fig. 5), from which a significant energy barrier of 88 K was extracted. The range of *α* values from the Cole-Cole (Argand) plot (Fig. 5, lower panel, inset) suggested discrimination between the differing Dy^III^ environments in the crystal structure, though only one maximum in *χ*″ was seen. From the Arrhenius plot (Fig. 5, lower panel, main), ln *τ* was temperature-dependent down to 2.5 K, suggesting quantum tunnelling of magnetisation below this temperature; curiously this was very inefficient, as evidenced by the lack of increase in *χ*″ in field, when compared to other lanthanide compounds [21]. **(Tb-5)** displayed the opposite behaviour, with a significant QTM and consequent lack of *χ*″ maxima in zero-field. This unusually inefficient, but desirable, QTM of **(Dy-5)** was then explored in some detail using CASSCF/RASSI/SINGLE_ANISO calculations by Ungur and Chibotaru on {Co^III^_2_Dy^III^Lu^III^} models [20].

QTM is effectively suppressed in the following manner: the ground doublet has a small splitting (10^−6^ cm^−1^), due to the non-Kramer's state resulting from two coupled Dy^III^ ions, which reduces QT. A second reason is the non-magnetic ground state, from strong dipolar coupling, which reduces the transverse influence from neighbouring molecules.

In modelling the magnetic data, the exchange postulated in **(Gd-5)**, *i.e.* zero, was also indistinguishable from models where it was finite, taking into account the effects of zero field splitting (ZFS), meaning that this technique could not confirm the presence or otherwise of an interaction.

Further magnetic measurements with the doped Dy^III^:Y^III^ compounds **(Dy:Y-5)**, where the percentage ratios were altered, indicated that the SMM behaviour of **(Dy-5)** was a single-ion effect and that the exchange between ions is indeed the source of the reduced QTM, being less prominent as the Dy^III^—Dy^III^ contribution is removed.

Rinehart et al. [22] have also seen much reduced QTM in a [Ln^III^_2_N_2_(NSiMe_3_)_4_(THF)_2_]^−^ compounds, where Ln^III^ is Tb^III^ or Dy^III^, bridged by a radical N_2_^3−^•ligand. The unusually strong coupling between lanthanide ions, several orders of magnitude larger than is normally found, appeared to be the key to obtaining the large hysteresis temperatures, without zero-field relaxation, though **(Dy-5)** would suggest qualitatively similar results can be obtained without resorting to such a “radical” approach. This reduction of QT by exchange-biasing has been well explored for 3d-transition metals, but is poorly understood in 4f compounds at present.

### 2.5 Developments

More recently, several variations on the {Co^III^_2_Dy^III^_2_} arrangement were discovered with teaH_3_ also by utilising acacH (acetylacetone). These [23], [24] are quite similar and formulated as [Co^III^_2_Dy^III^_2_(OMe)_2_(teaH)_2_(acac)_4_(NO_3_)_2_] **(6)**, [Co^III^_2_Dy^III^_2_(OH)_2_(teaH)_2_(acac)_4_(NO_3_)_2_]·4H_2_O **(7)** and [Co^III^_2_Dy^III^_2_(OMe)_2_(mdea)_2_(acac)_4_(NO_3_)_2_] **(8)**, with the now familiar butterfly arrangement of metals. The teaH ligands bond with the 3.2.2.0.1 mode, centred on an outer Co^III^ and the mdea^2−^ showing the 3.2.2.1 mode in the same way. Energy barriers, *U*_eff_, for each are single-ion in origin, and around 30 K, with more than one thermal process detected at higher applied frequencies of a.c. field.

More variations show the large effects of subtle changes in geometry and lanthanide: a.c. susceptibility measurements on [Co^III^_2_Ln^III^_2_(OH)_2_(bdea)_2_(acac)_2_(NO_3_)_4_], where Ln^III^ is Tb^III^ or Dy^III^ **(9)**, showed only the latter is an SMM, with a large *U*_eff_ of *ca*. 169 K and no pure QTM above 7 K. The bdea^2−^ bonds with the previously known 3.2.2.1 mode, being centred on Co^III^. Compared to **(6)** this energy barrier is increased by almost six times; that the enhancement arises from only a change in ligand is noted as a potentially useful way to tune the relaxation properties of existing compounds.

### 2.6 Strong lanthanide(III) dependence: {Mn^III^_2_Ln^III^_2_} and {Mn^III^_2_Ln^III^_3_}

**Fig. 6:**
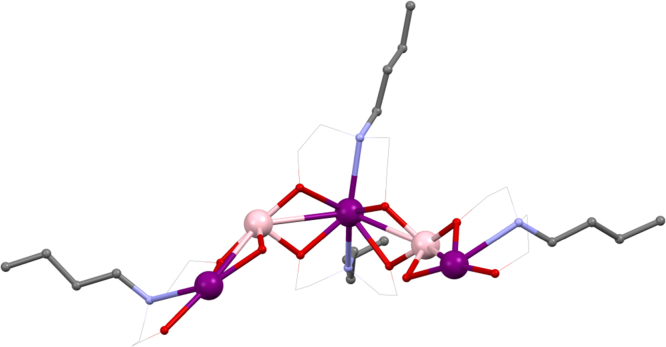
Skeletal view of [Mn^III^_2_Ln^III^_3_(^*n*^BudeaH)_3_(^*n*^Budea)_2_(O_2_C^*t*^Bu)_8_] **(11)**. Key: Ln^III^, purple spheres; Mn^III^, pale pink; O, red spheres (^*n*^Budea(H)); N, blue spheres (^*n*^Budea(H)); C, grey wireframe and spheres for R groups; no H atoms, solvent, or carboxylate groups are shown for clarity See Ref. [26] for structural information.

Using ^*n*^BudeaH_2_, the strong influence that the choice of lanthanide can have on the structure of coordination compounds was revealed. [Mn^III^_2_Ln^III^_2_(^*n*^Budea)_2_(^*n*^BudeaH)_2_(O_2_C^*t*^Bu)_6_]·2MeCN **(10)** [25] and [Mn^III^_2_Ln^III^_3_(^*n*^BudeaH)_3_(^*n*^Budea)_2_(O_2_C^*t*^Bu)_8_]·MeCN **(11)** [26] (shown in Fig. 6, where for clarity Mn^III^ is shown in pale pink to distinguish it from a Ln^III^), where HO_2_C^*t*^Bu is pivalic acid, are prepared from the same reaction; Mn^II^(O_2_CCH_3_)_2_·4H_2_O, pivalic acid, ^*n*^BudeaH_2_ and Ln^III^(NO_3_)_3_·*n*H_2_O being stirred in acetonitrile.

Depending on the size of the lanthanide used, one of the two products forms: **(10)** from La^III^, Ce^III^, Pr^III^ or Nd^III^ or **(7)** from Tb^III^, Dy^III^, Ho^III^, Er^III^ or Y^III^. The former has an incomplete double cubane structure, with slight changes to those previously seen. The outer metals are lanthanides, (*cf.* **(5)**) linked to one of the inner Mn^III^ ions by a pivalate group and all metals have one amine-diol ligand centred on them by N-bonding; for Ln^III^ this is ^*n*^BudeaH^−^, with the mode 2.2.1.1, the alkoxy arms linking Ln^III^ and Mn^III^ and capping the former. For each Mn^III^ the diol is ^*n*^Budea^2−^, bonding with the 4.3.2.1 mode. One arm links three metals in a Mn^III^_2_Ln^III^ triangle and the other links a Mn^III^ and Ln^III^. The remaining pivalate groups bond with 1.1.1 and 1.1.0 modes to each lanthanide. BVS and geometric analysis assigned the Mn^III^ oxidation states in this pair of compounds.

Given that the coordination number increases upon moving from the heavier, smaller lanthanides to the lighter, larger, metals, from eight to nine, this may explain the resulting change in topology, ligands moving to accommodate these preferences. However, given that the compounds above, *e.g.* **(2)** feature nine-coordinate Dy^III^, this cannot be the only factor. The relatively bulky butyl “tail” of the diethanolamine ligand in **(11)**, relative to teaH^2−^ in **(2)** may block the extra site from becoming involved.

**(11)** has a “horse-shoe” topology made up of alternating 3d and 4f metals. Rooted with an N-donor to the central Ln^III^ are two 3.2.2.1 ^*n*^Budea^2−^ ligands, both linking this metal to adjacent Mn^III^ ions. Pivalate groups bridge the four pairs of adjacent metals in 2.1.1 fashion and cap the terminal lanthanides in both a 1.1.0 and 1.1.1 manner. This leaves the ^*n*^BudeaH^−^ ligands that link the outer-most metals on each end and cap the lanthanide with a protonated arm, overall being 2.2.1.1. The third ^*n*^BudeaH^−^ links across the end of the “horse-shoe” with the 4.2.2.0 mode, a rare example where the N-atom is unbound. The sharp break with lanthanide size again suggests this is an important factor, the “break” usually occurring around Gd^III^, though this metal ion was not reported for either **(10)** or **(11)** here. Each lanthanide(III) in **(11)** has a distorted eight coordinate {N_2_O_6_} square-antiprismatic geometry, where the Ln^III^—N bonds are significantly longer than those to oxygen atoms, *ca*. 2.7 *versus* 2.3 Å.

Qualitatively similar magnetic behaviour in *χT*(*T*) was found for both of **(10)** and **(11)**, showing a decrease at lower temperatures, after expected values were found at room temperature for uncoupled ions. Furthermore the non-saturation and overlapping of magnetisation curves suggested anisotropy in each case, presumably at least partly due to the Mn^III^, because of its presence in the La^III^ and Y^III^ compounds, the (approximately) aligned Jahn–Teller axes in **(6)** likely contributing. These latter two were available to give information on the magnetic interactions between the Mn^III^ ions. For the former this was **(La-10)** and, using Eq. (1), *J* = 5.0 K when *g* = 2.12.

For **(Y-11)**, using Eq. (2) gave *D*/*k*_*B*_ = 4.2 K, *g* = 2.074 and with *zJ*′/*k*_*B*_ = 0.055 K, the latter value from the mean-field approximation, indicating a large anisotropy and weak coupling. The anisotropy here must be due to the aligned JT axes of the Mn^III^ ions.

(2) $$\hat{H}=D[({\hat{S}}_{z}^{2}-1/3\hat{S}(\hat{S}+1)]+g\mu_{\beta }H\hat{S}$$

*χ*″ frequency dependence was found in **(Ce-10)**, **(Nd-10)**, **(Tb-11)** and **(Dy-11)**, though only for **(Nd-10)** could *U*_eff_ be determined above 1.8 K and with *ν* = 1500 Hz, as 10 K.

Overall, the lanthanide ions must play a role, as not all compounds exhibit this dependence, though most likely this will be in concert with the Mn^III^ ions, given the *D*/*k*_*B*_ = 4.2 K observed in **(Y-11)**. Elsewhere the Dy^III^ compound in isostructural series gives the largest barrier, though here this does not show maxima in *χ*″, which should motivate further investigations.

### 2.7 Lanthanides only: {Ln^III^_4_}

**Fig. 7:**
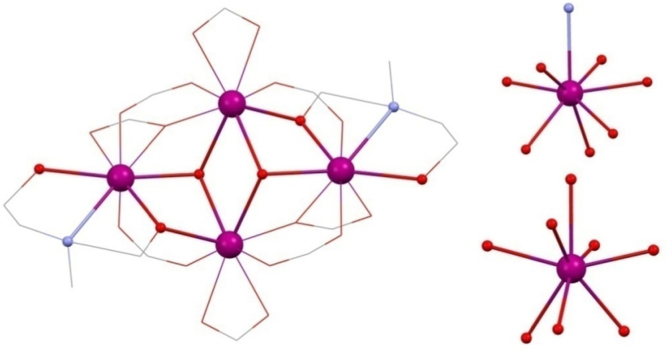
Left, [Ln^III^_4_(OH)_2_(mdeaH)_2_(O_2_C^*t*^Bu)_8_] **(12)**. Right, geometries of the outer Ln^III^, top, and inner Ln^III^, lower. Key: Ln^III^, purple spheres; O, red spheres (mdeaH) and wireframe (OH and pivalate); N, blue spheres (mdeaH); C, grey wireframe; no H atoms or ^*t*^Bu groups are shown for clarity. See Ref. [27] for structural information.

The first and so far only reported RdeaH_2_ 4f butterfly was [Ln^III^_4_(OH)_2_(mdeaH)_2_(O_2_C^*t*^Bu)_8_] **(12)** [27] (Ln^III^ = Tb^III^, Dy^III^, Ho^III^, Er^III^ or Tm^III^ and mdeaH_2_ is methyldiethanolamine) (shown in Fig. 7, left). The presence of lanthanides in all sites evidently requires higher coordination numbers to be filled compared to 3d–4f compounds which, along with the reduced coordinating ability of the amine-diol used here compared to an amine-triol requires more co-ligands. mdeaH^−^ bridges between two metals with one arm and caps with its other donors onto the same metal in 2.2.1.1 fashion, this being mirrored on the remaining metals. Four pivalate groups frame the core with the 2.1.1 mode and two cap the central metals with the 1.1.1 mode. The remainder bridge between pairs of metals that are not linked by the diol, with a 2.2.1 mode. The simplicity of the synthesis, combining Dy^III^(NO_3_)_3_·*n*H_2_O, pivalic acid and mdeaH_2_ in acetonitrile, suggests many variations to explore.

**Fig. 8:**
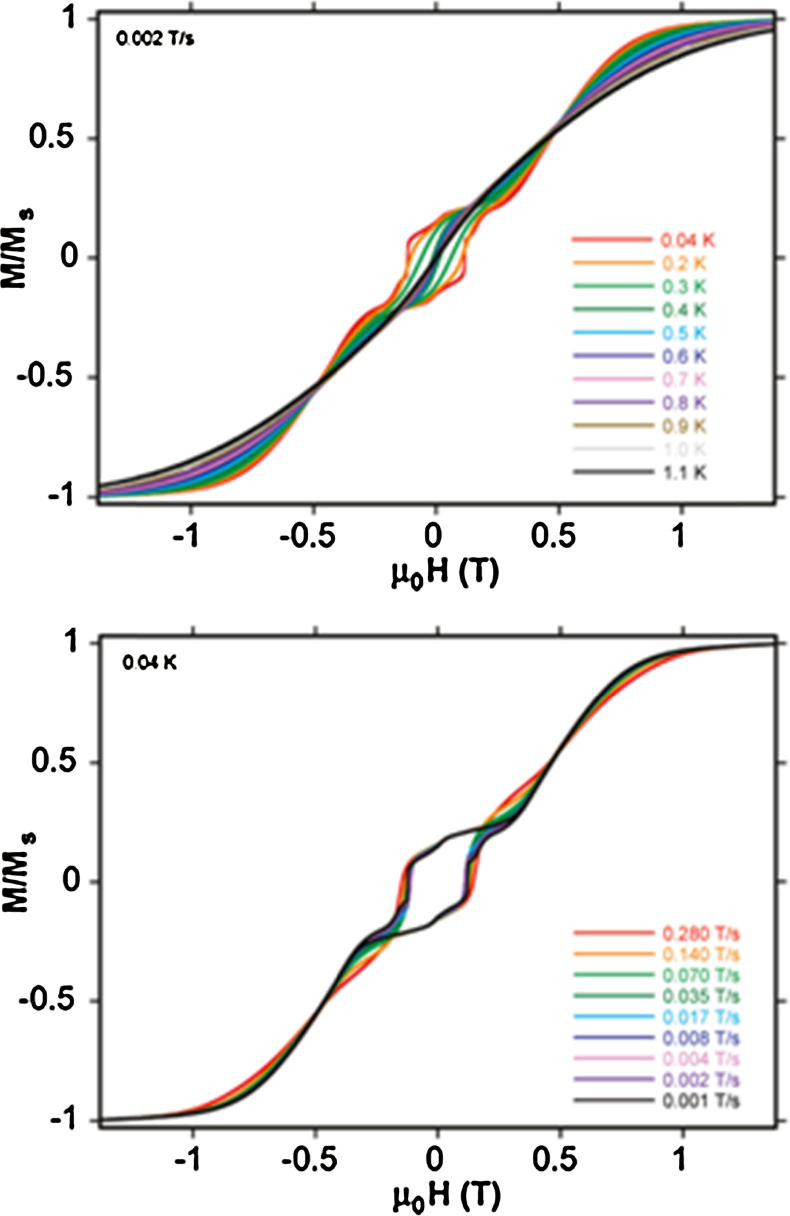
Hysteresis loops for [Dy^III^_4_(OH)_2_(mdeaH)_2_(O_2_C^*t*^Bu)_8_] **(Dy-12)**. Upper panel: measured on single-crystals at 0.002 T s^−1^ for various temperatures. Lower panel: measured at 0.04 K at various sweep rates between 0.001 and 0.280 T s^−1^.

Adapted with permission from [27]. Copyright (2010) American Chemical Society.

Previous examples of this topology have shown large energy barriers, notably [Dy^III^_4_(OH)_2_(bmh)_2_(msh)_4_Cl_2_] [13], where bmhH_2_ is 1,2-bis(2-hydroxy-3-methoxybenzylidene)hydrazone and mshH is 3-methoxysalicylaldehyde hydrazone. *U*_eff_ was 170 K and hysteresis loops open above 7 K. For **(Dy-12)** a more modest 6.2–6.9 K energy barrier was observed, increasing upon application of a magnetic field. Hysteresis loops are seen below 1.1 K (Fig. 8, upper panel) which have steps, both these features indicating resonant QTM, as this feature is absent under zero-field. There is also a slight dependence on sweep rate, confirmation is in the lower panel of Fig. 8.

One possible explanation for this discrepancy in the magnitude of *U*_eff_ lies in the much more regular geometries found around the Dy^III^ ions in [Dy^III^_4_(OH)_2_(bmh)_2_(msh)_4_Cl_2_], whereas these are rather distorted from square-antiprismatic in **(12)**. In fact there are two types, both eight coordinate, and described as distorted dodecahedral, and shown in Fig. 7, right.

A more axial geometry appears, in general, in those compounds with the highest *U*_eff_ values, namely Blagg et al.’s {Ln^III^_5_} series [28] and Ishikawa's [Ln^III^Pc_2_]^−^ series, where H_2_Pc is phthalocyanine [8], which seems to favour the stabilisation of the highest magnitude *m*_*J*_ states relative to others, so creating a large thermal barrier to relaxation, as demonstrated by Rinehart and Long [29].

### 2.8 Thoughts on incomplete double cubanes

The formation of teaH_3_ and RdeaH_2_ butterflies has been reported for combinations of heavier lanthanides and Fe^III^, Mn^III^ and Co^III^ using the teaH_3_ pro-ligand. This has involved room temperature reactions where components of acid, amino alcohol and lanthanide nitrate are reacted at room temperature, or with gentle heat. In **(5)** the acid was supplied as a pro-ligand instead of as the carboxylate *via* a 3d metal triangle with the effect that Co^III^ was on the outer position of the compound. Perhaps there is a greater competition for the inner site, and the lanthanide is better protected there, so wins out, requiring this due to its higher coordination number. Only in **(5)** was base added, being required to deprotonate the acid, with the effect that we do not see a fully deprotonated teaH_3_ ligand in any of compounds examined so far. This leads to somewhat reduced coordination modes compared to those seen for tea^3−^, *e.g.* 7.3.3.3.1 in **(19)** and 4.2.2.2.1 for **(25)**–**(26)**, *vide infra*. For the RdeaH_2_ compounds **(12)** a similar synthesis was used, with the available ligands seemingly accommodating the reduced bonding of this ligand *cf.* teaH^2−^. The most unusual set of compounds here are **(10)** and **(11)** with such a large structural change, depending on the lanthanide used. Unfortunately this exploration across the 4f series has not been tested for the others in this set of butterflies **(1)**–**(10)**, only the “magnetically interesting” compounds being reported, so conclusions cannot be extrapolated.

Already we can see how these flexible ligands have stabilised structures with different, albeit similar sized, transition metals, including by modifying their own state of protonation. Furthermore different structures across the lanthanide series were stabilised, accounting for the change in size from La^III^ to Er^III^.

We now turn to larger ring structures and the even richer variety of bonding modes that they display.

## 3 Metallo-rings

### 3.1 Go large: {Fe^III^_16_Ln^III^_4_}

**Fig. 9:**
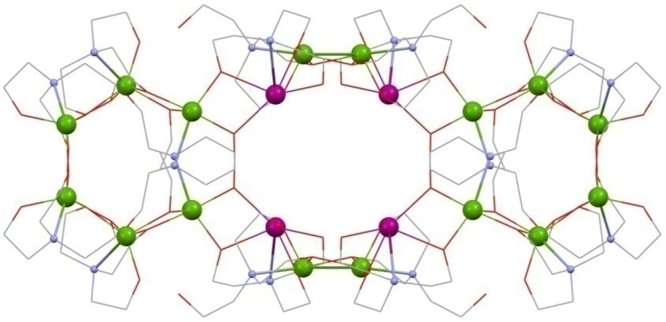
[Fe^III^_16_Ln^III^_4_(tea)_8_(teaH)_12_(O_2_CCH_3_)_8_] **(13)**. Key: Ln^III^, purple spheres; Fe^III^, green; O, red wireframe; N, blue spheres (tea^3−^ and teaH^2−^); C, grey wireframe; no H atoms or acetate groups are shown for clarity. See Ref. [30] for structural information.

The largest 3d–4f cage yet discovered is the metallo-ring [Fe^III^_16_Ln^III^_4_(tea)_8_(teaH)_12_(O_2_CCH_3_)_8_](NO_3_)_4_·16H_2_O·*y*MeCN **(13)** [30] (Ln^III^ = Sm^III^, Eu^III^ or Gd^III^, *y* = 11, and when Dy^III^, Tb^III^ or Ho^III^, *y* = 10) shown in Fig. 9. The synthesis is very similar to that of the incomplete double cubanes above, despite obvious topological differences, though there appears a direct correlation between the smaller carboxylate ligand with a larger resultant structure.

The {Fe^III^_6_} horse-shoes at either end of the structure, the whole of which could be thought of as two {Fe^III^_6_Ln^III^_2_} rings linked by two {Fe^III^_2_} chains, is held together by four 3.2.2.1.1 tea^3−^ ligands, which are centred on each of the four inner metals and connect them to their two neighbours; this is a ubiquitous mode, though the only one found so far for tea^3−^, rather than teaH^2−^, in 3d–4f chemistry and is unusual because an O-donor is only bonding terminally, though there is some partial H-bonding to other OH functionalities. The occurrence of this mode across numerous topologies indicates co-ligands play a significant role in deciding the shapes of the metal cores, expectedly, since the amino-alcohols are flexible.

The two Fe^III^ ions on the end of this ring are linked to one Ln^III^ by a 3.2.2.0.1 teaH^2−^ ligand, the protonated arm being unbound. The terminal Fe^III^ and Ln^III^ are also bridged by an acetate group 2.1.1, linking to a different Fe^III^, one of a bridging dimer on either side of the structure by a 3.2.2.1.1 teaH^2−^, centred on the 4f metal. These 4f ions are also linked by an acetate group to the equivalent ion on the opposite side, forming two sets of cross-links (not shown) and so have a dodecahedral eight coordinate geometry, though this has not precluded SMM behaviour in the past. The key may be in the alignment and cancelling of the Dy^III^ anisotropy axes, which are not co-parallel.

The two Fe^III^ ions of each bridging dimer are linked by an acetate ligand and to adjacent lanthanides on either side by two 3.2.2.0.1 teaH^2−^ ligands, so linking the two halves. **(13)** demonstrates the versatility of the triol ligand, which shows three distinct bonding modes depending on its form and to which metals it bonds.

In the six cases investigated, antiferromagnetic interactions were dominant, again signified by decreasing *χT* products with temperature. The use of **(Eu-13)** and **(Sm-13)** enabled an *S* = 0 ground state to be deduced as Eu^III^ becomes diamagnetic at the lowest temperatures investigated and the even number of Fe^III^ spins cancel out, hence antiferromagnetic Fe^III^ exchange was postulated.

Based on Mössbauer investigations of **(Dy-13)** the Dy^III^—Fe^III^ interaction was very small and the Fe^III^ exchange dominates the magnetic behaviour.

### 3.2 Octa-ring: {Cr^III^_4_Dy^III^_4_}

The use of Cr^III^ is rather rare in 3d–4f molecular magnetism though recently has become a focus for isotropic magnetic refrigerants [31]. A beautiful example in amine-diol chemistry is the “square-in-butterfly” [Cr^III^_4_Dy^III^_4_(OH)_4_(mdea)_4_(N_3_)_4_(O_2_C^*t*^Bu)_8_]·3CH_2_Cl_2_ **(14)** [32], from the reaction of Dy^III^(NO_3_)_3_·*n*H_2_O, pivalic acid, methyldiethanolamine (mdeaH_2_), NaN_3_ and Cr^II^Cl_2_ in dichloromethane under an inert atmosphere, the oxidation of the transition metal taking place upon exposure to air. **(14)**, shown in Fig. 11 as its otherwise identical Mn^III^ cousin, *vide infra*, where Jahn–Teller effects will be discussed, is composed of four linked {Cr^III^Dy^III^_2_} units. These are themselves linked by a 3.2.2.1 mdea^2−^ ligand, this being centred on the Cr^III^ ion, and two pivalates that bridge between a lanthanide and two 3d metal neighbours as 2.1.1. N_3_ groups and OH groups link adjacent lanthanides into an inner square, these lying in one plane, with a {Cr^III^_4_} butterfly lying above (two ions) and below (two more).

Extensive modelling and magnetic measurements were performed, the latter indicating anisotropy from non-superimposable magnetisation *versus* reduced field curves. From the Arrhenius plot, a *U*_eff_ value of 15 K was observed with hysteresis loops obtained below 1.1 K confirming this is unambiguously an SMM. From these data we infer the presence of quantum-tunnelling by their stepped nature, features absent in measurements above 1.8 K. CASSCF/CASPT2 calculations were performed to obtain fits of the magnetisation data; for Dy^III^ a satisfactory fit used the parameters *g*_*x*_ = 1.7, *g*_*y*_ = 5.8 and *g*_*z*_ = 14.4, and a negative *J* value, indicating antiferromagnetic exchange between Cr^III^ and other metal ions.

**Fig. 10:**
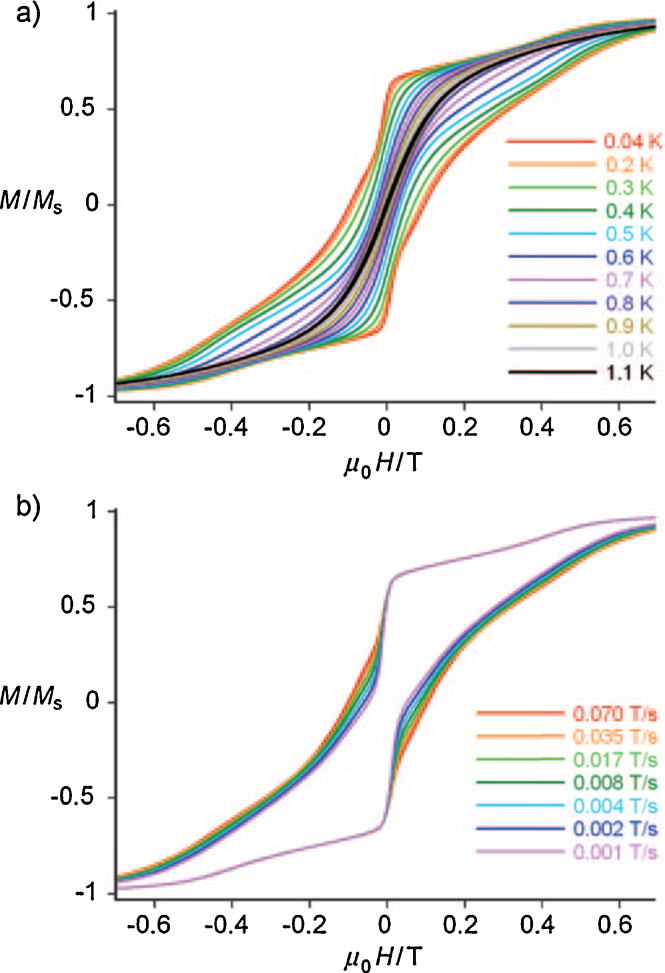
Upper panel: Hysteresis loops on single crystals of [Cr^III^_4_Dy^III^_4_(OH)_4_(mdea)_4_(N_3_)_4_(O_2_C^*t*^Bu)_8_]·3CH_2_Cl_2_**(14)** at a 0.035 T s^−1^ sweep rate between 0.04 and 1.1 K. Lower panel: Loops for a fixed temperature of 0.04 K for sweep rates between 0.001 T s^−1^ and 0.07 T s^−1^.

Reproduced with permission from [32]. Copyright 2010, Wiley-VCH Verlag GmbH & Co. KGaA.

These significant transverse *g*_*x*_ and *g*_*y*_ values also contribute to significant QT. The avoided crossings observed in the energy spectrum for **(10)**, are in agreement with the observed quantum tunnelling steps in the single-crystal hysteresis data, shown in the upper panel of Fig. 10, for the variable temperature experiment, with the sweep dependent curves shown in the lower panel.

Both coordination and site symmetries play an important role in the SMM: Cr^III^ is basically isotropic, owing to the octahedral geometry at each site but the Dy^III^ ions, with *D*_2d_ site symmetry, have significant anisotropies which have a none-zero sum.

### 3.3 Ringing the changes: {Mn^III^_4_Ln^III^_4_}

**Fig. 11:**
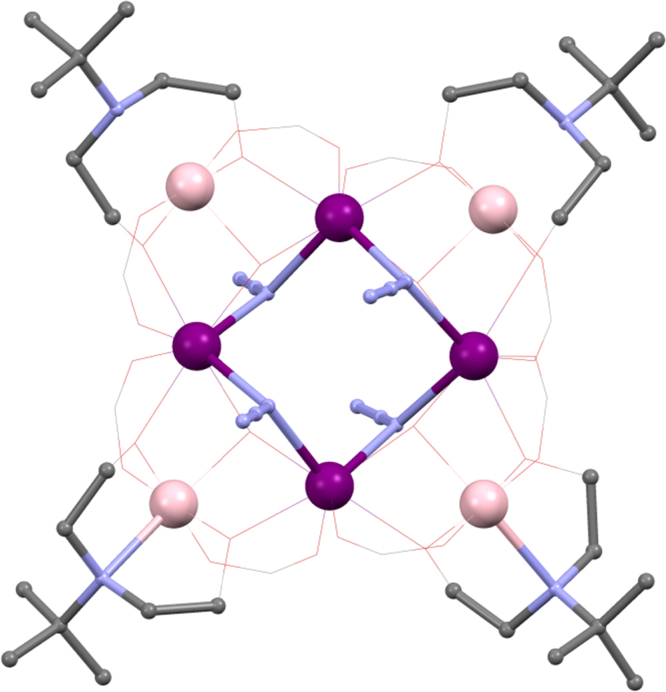
[Mn^III^_4_Ln^III^_4_(OH)_4_(^*t*^Budea)_4_(N_3_)_4_(O_2_C^*t*^Bu)_8_] **(15)**. Key: Ln^III^, purple spheres; Mn^III^, pink; O, red wireframe; N, blue spheres; C, carboxylates grey wireframe, spheres for ^*t*^Budea C atoms; no H atoms or carboxylate ^*t*^Bu groups are shown for clarity. See Ref. [33] for structural information.

Using modifications of the above procedure, several close Mn^III^ analogues of **(14)** were synthesised, though requiring ^*t*^BudeaH_2_, tert-butyl diethanolamine, and a selection of co-ligands and solvents. Formulated as [Mn^III^_4_Ln^III^_4_(OH)_4_(^*t*^Budea)_4_(X)_4_(O_2_C^*t*^Bu)_8_]·solvent **(15)** [33] (Ln^III^ is Y^III^, Eu^III^, Gd^III^, Tb^III^, Dy^III^ or Ho^III^, X = N_3_^−^; with Dy^III^ X may also be ^−^OCN; and, where X = NO_3_^−^, Gd^III^, Tb^III^, Dy^III^, Ho^III^ and Er^III^ analogues can be made with solvent being toluene or acetonitrile). The changing of the N_3_ bridges from those seen in **(10)** does not change the overall structure, given in Fig. 11, though a change in proligand from mdeaH_2_ to ^*t*^BudeaH_2_ is required.

All Dy^III^ and Tb^III^ compounds showed out of phase *χ*″ frequency dependence, though energy barriers could not be obtained in the absence of maxima, invariance to applied d.c. fields, though, suggested QTM is absent. The lanthanide(III) ions are the source of this, as the anisotropy axes, arising from a Jahn–Teller elongation about Mn^III^ will approximately cancel. The change in the amine-diol ligands between **(14)** and **(15)** for the Dy^III^ compounds should not significantly alter the magnetic properties in general, though the SMM behaviour are different. Further results may be needed to elucidate the reasons why.

### 3.4 Saddle-up: {Fe^III^_4_Dy^III^_4_}

The pair of structurally related {Fe^III^_4_Dy^III^_4_} and {Mn^III^_4_Ln^III^_4_} saddle-compounds were prepared using distinct amine-triol and diols, respectively, indicating a robust topology, which though superficially similar to **(13)** and **(14)**, are quite distinct.

An octametallic “saddle”, incorporating the N_3_ co-ligand, was made by allowing a refluxed solution of Dy^III^Cl_3_·6H_2_O, Fe^III^Cl_3_, NaN_3_ and teaH_3_ in methanol:acetonitrile (1:2) to stand for 3 days, giving crystals of [Fe^III^_4_Dy^III^_4_(teaH)_8_(N_3_)_8_(H_2_O)]·4MeCN·H_2_O **(16)** [34]. This is made up of alternating 3d and 4f ions, with one of the Dy^III^ sites distinct by the bonding of a water molecule. The 4f ions are linked to their adjacent 3d neighbours by two teaH^2−^ ligands, on the inside and outside of the saddle (3.2.2.1.1 mode). H-bonding is extensive and is seen between each of the two N_3_ ligands bound to each Fe^III^ and the OH arm of teaH^2−^, and between the solvent water molecule protons and the O of teaH^2−^.

This structure is remarkable for its lack of large co-ligands, though its similarity to those seen above suggests these are not a significant factor, as long as coordination numbers can be filled. The closed structures (rings and cubanes and partial chains) seen so far may be expected, as an organic coating encloses a metal core.

The isotropy of the octahedral Fe^III^ simplifies the analysis of magnetic data: ferromagnetic interactions are implied by *χT*(*T*) measurements which, with magnetisation measurements, implicate anisotropy, likely due to Dy^III^, as supported by Mössbauer studies. The capped square-antiprismatic geometry is theoretically not ideal for SMM behaviour, though, this compound is in fact an SMM, as shown by both a frequency dependence of *χ*″ below 2.8 K and a temperature dependent regime in the Arrhenius plot, corresponding to an energy barrier of 30.5 K. SMM behaviour is confirmed by the presence of smooth hysteresis loops below 1.4 K, and so also show the lack of a QTM.

### 3.5 Keep on riding: {Mn^III^_4_Ln^III^_4_}

**Fig. 12:**
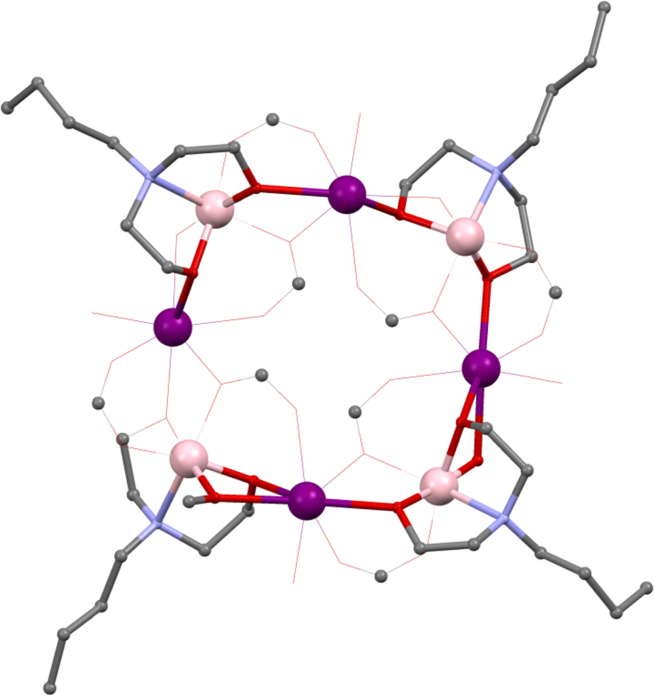
[Mn^III^_4_Ln^III^_4_(^*n*^Budea)_4_(O_2_CH)_4_(OMe)_4_(O_2_CEt)_4_(O_2_CEt)_4_(MeOH)_4_] **(13)**. Key: Ln^III^, purple spheres; Mn^III^, pale pink; O, red spheres (^*n*^Budea^2−^) and wireframe (OH and carboxylates— note only the inner ligands are therefore really formates); N, blue spheres (^*n*^Budea^2−^); C, grey wireframe and spheres for carboxylate bridges and ^*n*^Budea; no H atoms or Et groups are shown for clarity. See Refs. [35], [36] for structural information.

[Mn^III^_4_Ln^III^_4_(^*n*^Budea)_4_(O_2_CH)_4_(OMe)_4_(O_2_CEt)_4_(O_2_CEt)_4_(MeOH)_4_] **(17)** [35] (Ln^III^ = Gd^III^ or Dy^III^) was synthesised using the lightest carboxylate, ^−^O_2_CH, by combining Mn^II^(O_2_CEt)_2_, sodium formate, Ln^III^(NO_3_)_3_·*n*H_2_O and ^*n*^BudeaH_2_ in methanol. An alternative preparation [36] gave the analogues with Sm^III^, Tb^III^, Ho^III^, Er^III^ and Y^III^. Similar in its saddle structure to **(16)**, and shown in Fig. 12, this has alternating 3d and 4f ions where the diol bridges across an N-bonded Mn^III^ and adjacent Ln^III^ ions with a 3.2.2.1 mode.

One methanol bonds to each of the latter, and a methoxy group bridges between distinct hetero-metal pairs. The presence of two different carboxylates here and three distinct bonding modes is uncommon; an ethanoate bonds 1.1.0 to each 4f metal and bridges distinct hetero-metal pairs. Formate bonds with the 3.2.1 mode between two lanthanides and one Mn^III^. All of the 3d ions can be viewed in a plane, a difference to **(11)** that has a more marked distortion. Both compounds show decreasing *χT* products with decreasing temperature, which could be assigned to anti-ferromagnetic coupling for **(Gd-17)** and possibly a combination of this and anisotropy effects for **(Dy-17)**. Only the latter of these two compounds is an SMM, though, suggesting the importance of the Ln^III^ ion, with *U*_eff_ = 12 K. The stepped micro-SQUID hysteresis loops below 0.5 K indicate QTM is important at these low temperatures also confirming this as a true SMM. Later, SMM behaviour was observed in Sm^III^, Tb^III^ and Y^III^ analogues, the last rather interestingly as this must arise from the Mn^III^ ions only, despite the alignment of their JT axes, as above, meaning that their anisotropies must almost cancel. Furthermore, SMM behaviour was curiously absent in the {Mn_4_^III^Gd_4_^III^} compound, which implies the anisotropy of the lanthanide(III) ions is playing a major role, despite the lack of a fine-tuning for each 4f metal, these being in a common environment.

### 3.6 The free-wheeling: {Ln^III^_6_}

**Fig. 13:**
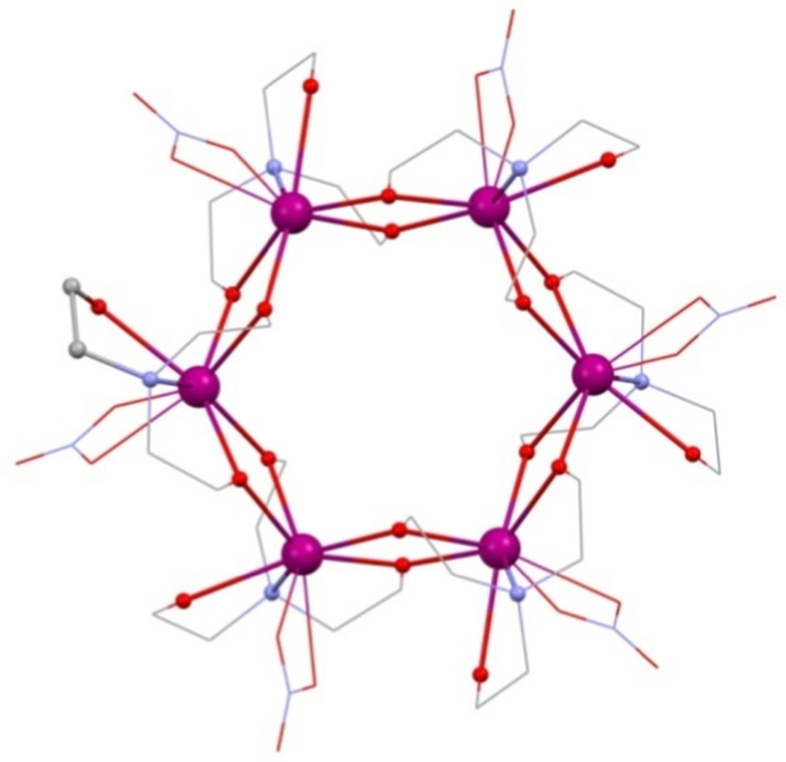
[Ln^III^_6_(teaH)_6_(NO_3_)_6_] **(18)**. Key: Ln^III^, purple spheres; O, red spheres (tea^3−^) and wireframe (NO_3_); N, blue spheres (teaH^2−^) and wireframe (NO_3_); C, grey wireframe; no H atoms or solvent molecules are shown for clarity. See Ref. [37] for structural information.

The intriguing [Ln^III^_6_(teaH)_6_(NO_3_)_6_]·8MeOH **(18)** [37], [38] (Ln^III^ = Gd^III^ or Dy^III^) wheel, shown in Fig. 13, is formed by layering ether upon a solution of Ln^III^(NO_3_)_3_·*n*H_2_O, triethylamine and teaH_3_ in MeOH. Remarkably, these small ligands are able to stabilise a hexametallic core by coating inside and outside the metals. teaH^2−^ bonds across three lanthanides by linking adjacent metals to a central ion, then capping this with an OH arm and N, as is now familiar (3.2.2.1.1 mode), alternately above and below the plane. An H-bonded network is formed through the OH proton of teaH^2−^ to the solvent MeOH O-atom through further solvents to a different teaH^2−^ H-atom, so arranging three wheels. The wheel topology is rare in lanthanide chemistry, though examples of {Ln^III^_10_} have been previously reported [39]. This is also one of only two polymetallic 4f teaH^2−^ compounds currently published, though the unusual SMM properties surely motivate further interest. However, the probable antiferromagnetic coupling in **(Gd-18)**, which showed decreasing *χT* at low temperatures and slow magnetisation rates, would likely be a hindrance to a large magnetocaloric effect at low applied fields. This behaviour was projected to **(Dy-18)**, which showed intriguing SMM behaviour; the *χ*″ plots being divergent at low temperatures, though no maxima were seen down to 2 K. The understanding of the magnetism of this compound was later expanded upon in an *ab initio* theoretical study that revealed, amongst other details, a non-magnetic, toroidal, ground state; *i.e.* the anisotropy axes of Dy^III^ ions lie in the plane of the wheel, as was also seen in the celebrated {Dy^III^_3_} triangles of Powell [40]. This arrangement is more perfectly realised here and so the ground state spin is zero, *i.e. S* = 0 under zero-field, due to the S_6_ symmetry of the molecule. An appreciable quantum tunnelling for each Dy^III^ was ascribed to the significant transverse *g*-components supporting the experimental discovery of SMM behaviour only at low temperatures. In practice, such an arrangement is described as ideal for a robust qubit, though requires NMR investigations to elucidate to energy barrier of the degenerate toroidal ground states.

### 3.7 Thoughts on metallo-rings

In these larger structures we find rather complex SMM behaviour. For instance, in **(14)** and **(15)** changing the 3d metal to Mn^III^ apparently turns off the SMM behaviour compared to the Cr^III^ analogue. **(17)** shows a remarkable intransigence to the lanthanide(III) ion, which appears the source of SMM behaviour considering its absence in the isotropic gadolinium(III) compound. Finally, **(18)** is one of a handful of toroidal spin examples with potentially fruitful technological applications.

## 4 Hepta-metallic discs

### 4.1 Cooler than thou?: {Mn^II^_3_Ln^III^_4_}

**Fig. 14:**
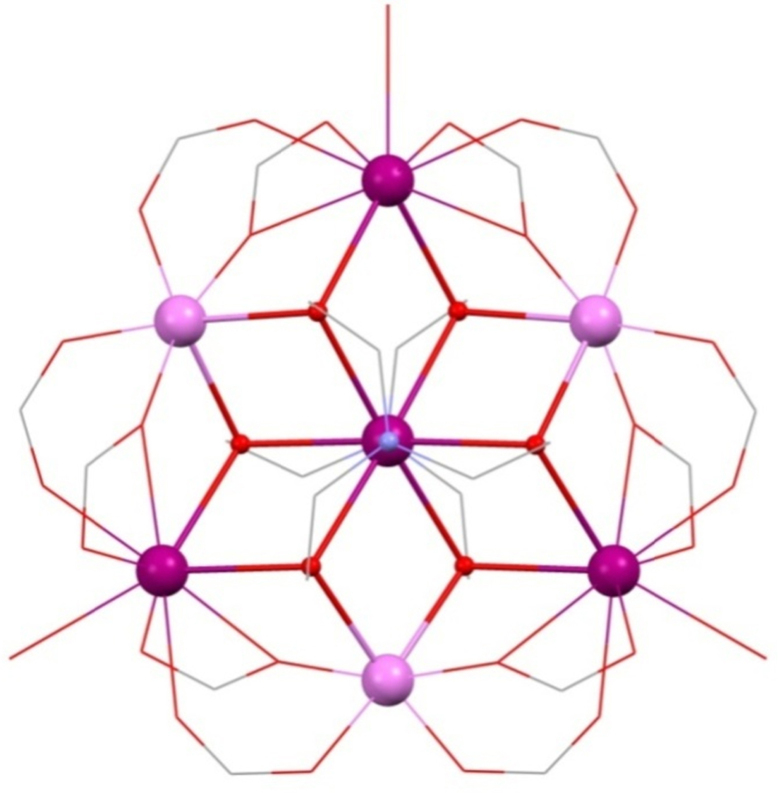
[Mn^II^_3_Ln^III^_4_(tea)_2_(O_2_CtBu)_12_(H_2_O)_3_] **(19)**. Right, geometries of the central Ln^III^, top, and outer Ln^III^, lower. Key: Ln^III^, purple spheres; Mn^II^, pale pink; O, red spheres (tea^3−^) and wireframe (H_2_O and pivalate); N, blue spheres (tea^3−^); C, grey wireframe; no H atoms or ^*t*^Bu groups are shown for clarity. See Ref. [46] for structural information.

Hepta-metallic compounds are relatively common in 3d chemistry with examples known for Mn, Fe, Co, Ni and Cu [41], [42], [43], [44], [45]. In 3d–4f and teaH_3_ chemistry, though, they are rare, there being only a recent single example, namely [Mn^II^_3_Ln^III^_4_(tea)_2_(O_2_C^*t*^Bu)_12_(H_2_O)_3_]·H_2_O **(19)** [46] (Ln^III^ = La^III^, Pr^III^, Nd^III^ or Gd^III^) as shown in Fig. 14. Amongst teaH_3_ and deaH_2_ compounds there are also very few prepared by solvothermal synthesis, as **(19)** is, though a common technique in molecular magnetism as a whole. Here, [Mn^III^_6_O_2_(O_2_C^*t*^Bu)_10_(4-Me-py)_2.5_(HO_2_C^*t*^Bu)_1.5_], where 4-Me-py is 4-methyl-pyridine, is combined with Ln^III^(NO_3_)_3_·*n*H_2_O, teaH_3_, triethylamine at 120 °C in acetonitrile, where Mn^III^ is reduced *in situ.*

The planar disc is made up of alternating outer 3d Mn^II^ (confirmed by BVS) and 4f metals, around a central Ln^III^ hub. A tea^3−^ ligand is centred above and below on this hub, bridging with all three arms in an extensive 7.3.3.3.1 mode, each arm bonding between {Ln^III^_2_Mn^II^} triangles, giving the most coordinating tri-alcohol so far.

Six pivalate groups bridge adjacent metals in 2.1.1 fashion around the edge of the disc, the remaining six with a 2.2.1 mode with one water molecule bound to each of the outer Ln^III^s. All Mn^II^ ions are octahedrally coordinated, so *S* = 5/2, which, along with the high spin, *S* = 7/2, and isotropy of Gd^III^, should make this compound a useful magnetic refrigerant, notwithstanding the general low densities of coordination compounds, a hindrance in that application.

Fitting the magnetic data of **(Gd-19)** with the anisotropic Hamiltonian (Eq. (3)), where *D* ≠ 0, gave *g* = 2.06, *D* = −0.011 cm^−1^, expectedly small, and a large ground state spin of *S* = 17/2, where *D* is axial zero-field splitting (ZFS) and *E* is rhombic ZFS, with no *J* value reported.

(3) $$\hat{H}=D{\hat{S}}_{Z}^{2}+E({\hat{S}}_{X}^{2}+{\hat{S}}_{Y}^{2})+g\mu_{\beta }S\cdot H$$

Separately, using **(La-19)**, and Eq. (4), below, the Mn^II^ exchange was reported as +0.2013 cm^−1^, *i.e.* ferromagnetic, where *g* = 1.99, fitted above 50 K, to prevent ZFS from interfering with the fitting to susceptibility data. These results may prove rewarding in the quest for SMMs if the planned oxidation of the Mn^II^ ions to Mn^III^ is successful.

(4) $$\hat{H}=-2J({\hat{S}}_{1}{\hat{S}}_{2}+{\hat{S}}_{1}{\hat{S}}_{3}+{\hat{S}}_{2}{\hat{S}}_{3})$$

What is most fascinating here is that the solvothermal technique results in the largest coordination mode yet found for a tea^3−^ ligand, 7.3.3.3.1. This behaviour was previously seen with other tripodal alcohol ligands to give more extensive bonding modes and may represent a future route to high performance MCE materials. The best materials require a low ligand to metal ratio so increasing the number of metals bound to each ligand could improve matters in this regard. Other examples, though show that this synthetic strategy is not generally applicable, though, *vide infra*.

## 5 Two to tango: dimeric 4f compounds

### 5.1 mdeaH_2_ and {Ln^III^_2_}

**Fig. 15:**
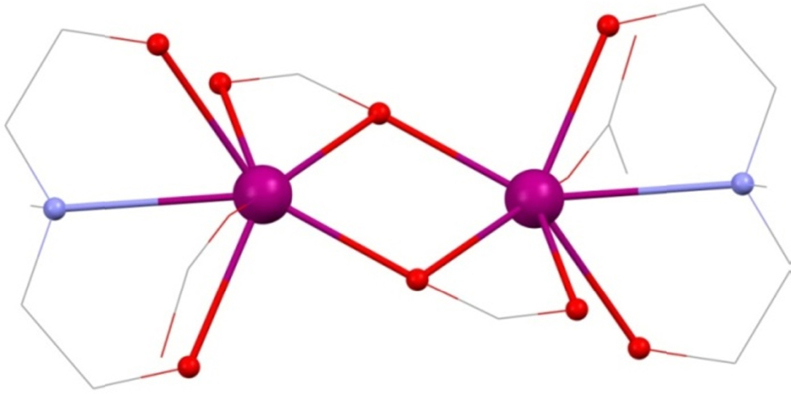
[Ln^III^_2_(mdeaH_2_)_2_(O_2_C^*t*^Bu)_6_] **(20)**. Key: Ln^III^, purple spheres; O, red spheres (mdeaH_2_ and bridging pivalates) and wireframe (terminal pivalates); N, blue spheres (mdeaH_2_); C, grey wireframe; no H atoms, ^*t*^Bu groups or 2.1.1 pivalates are shown for clarity. See Ref. [47] for structural information.

Dimeric structures represent simple compounds with which to investigate exchange interactions and there are three examples with amine-polyol ligands. The first is [Ln^III^_2_(mdeaH_2_)_2_(O_2_C^*t*^Bu)_6_] **(20)** [47] (Ln^III^ = La^III^, Ce^III^, Pr^III^, Nd^III^, Sm^III^, Eu^III^ or Gd^III^), shown in Fig. 15 in cutaway form, synthesised from refluxing of the appropriate Ln^III^(NO_3_)_3_·*n*H_2_O, mdeaH_2_ and pivalic acid in acetonitrile. This is made up of two pivalate bridged Ln^III^ ions, two ligands bonding with the mode 2.2.1, two with a 2.1.1 mode and one capping each of the ions as 1.1.0. The mdeaH_2_ ligand caps on each ion with the 1.1.1.1 mode where the protonated arm H-bonds to the terminal carboxylate O-atom. Therefore, this is more of a capping than bridging ligand, likely due to the lack of base added and the ligand remaining doubly protonated, which reduces the extent of the bonding. The magnetic data of **(Gd-20)** were fitted using the Hamiltonian given in Eq. (1) above, finding a best fit of *g* = 2.03 and *J* = 0.005 K, an extremely small ferromagnetic interaction, in line with an increase in *χT* at very low temperatures.

For the other analogues the decreasing *χT* with decreasing temperature was not fitted, but could be ascribed to either antiferromagnetic interactions, depopulation of Stark levels, or both.

### 5.2 H_3_sabhea and {Ln^III^_2_}

[Gd^III^_2_(sabheaH)_2_(NO_3_)_2_]·2MeOH **(21)** [48] was synthesised from methanolic solutions of Gd^III^(NO_3_)_3_·*n*H_2_O, sabheaH_3_ and NaOH where here the addition of base appears to assist in increasing the coordination mode, **(21)**. sabheaH_3_, N-salicylidene-2-(bis(2-hydroxyethyl)amino)ethylamine, is shown in Scheme 1, and comprises a functionalised RdeaH_2_, where the R group is C_2_H_4_NCHPh-*o*-OH. One amine-diol arm bridges the two metals with one arm bonding terminally and H-bonding to solvent methanol. The phenol—OH simply bonds terminally to one Ln^III^, its bulk seemingly assisting in enclosing the dimer from further aggregation, so overall the mode is 2.2.1.1.1. The coordination sphere of the metals is filled by a 1.1.1.0 nitrate. A decreasing *χT* product with decreasing temperature was assigned qualitatively to antiferromagnetic coupling and quantitatively to an exchange of *J*_12_ = −0.198 cm^−1^, *g* = 1.975, from the single *J* Hamiltonian, different to those seen previously, and given in Eq. (5).

(5) $$\hat{H}=-J_{12}{\hat{S}}_{1}\cdot {\hat{S}}_{2}$$

### 5.3 teaH_3_ and {Ln^III^_2_}

**Fig. 16:**
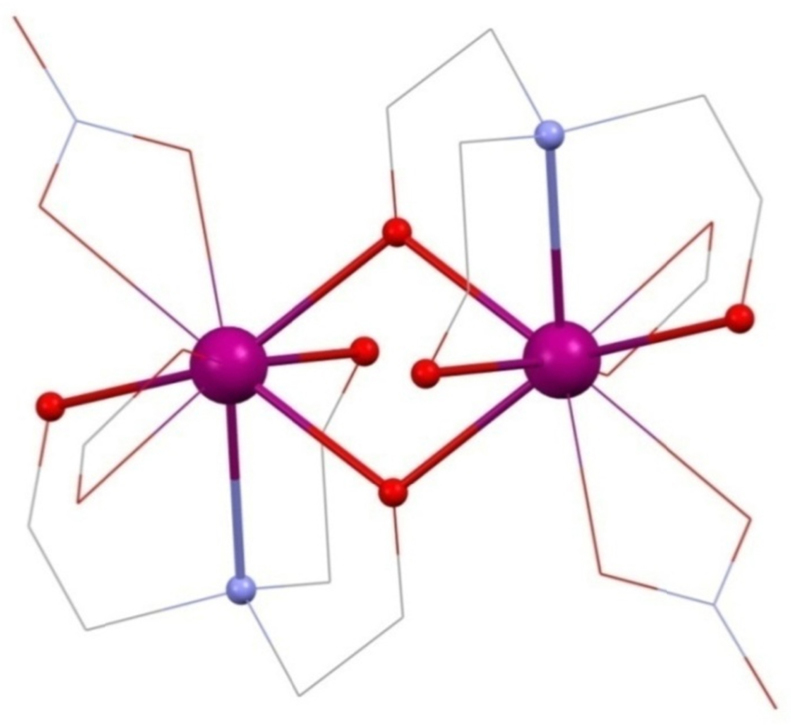
[Ln^III^_2_(teaH_2_)_2_(tpa)_2_(NO_3_)_2_] **(22)**, where tpaH is triphenyl acetic acid. Key: Ln^III^, purple spheres; O, red spheres (teaH_2_^−^); N, blue spheres; C, grey wireframe; no H atoms or CPh_3_ groups are shown for clarity. See future work [49] for structural information.

[Ln^III^_2_(teaH_2_)_2_(tpa)_2_(NO_3_)_2_] **(22)** [49] (Ln^III^ = Sm^III^, Gd^III^, Tb^III^, Dy^III^ or Ho^III^ and tpaH is triphenyl acetic acid), shown in Fig. 16, is formed from the solvothermal reaction at 100 °C of Ln(NO_3_)_3_·*n*H_2_O, teaH_3_, NEt_3_ and tpaH in acetonitrile. This type of synthesis is rare in teaH_3_ and RdeaH_2_ chemistry, despite the advantages of solubility and crystallinity that it can bring. The preparation is related to that previously reported for the {Ln^III^_7_} disc-like structures using the thmeH_3_ tri-alcohol ligand [50].

The metals are bridged by the deprotonated arm of teaH_2_^−^ and the protons assigned by charge balancing considerations. The remaining donor atoms bond solely to each metal, giving the 2.2.1.1.1 mode of bonding. Each ion is therefore nine-coordinate in a rather low symmetry environment, which accounts for the lack of SMM behaviour. The steric bulk of the triphenyl acetate, conceptually similar to the sabheaH_3_ employed in **(21)** may contribute to the relatively unusual bonding mode, both being rather restricted compared to other polymetallic compounds. The nitrate and tpa^−^ ligands cap both metals with two O atoms each.

Magnetic data were fitted using the Hamiltonian given in Eq. (1), finding a weak antiferromagnetic interaction of −0.114 cm^−1^, hindering the magnetocaloric response, which shows a maximum −Δ*S*_*M*_ value of *ca*. 20 J kg^−1^ K^−1^ (Δ*H* = 0–7 *T*, 3 K).

Exchange between metals is dependent on the bridging angle between metals, though for **(20)** there are also alternative exchange pathways other than through the amino-polyalcohol ligands, so this may be not be a straightforward analysis.

### 5.4 Thoughts: base *versus* bulk?

The above highlights an important synthetic challenge when using lanthanides. From our own failures and other reports, it is rather easy when using hydrated starting materials such as Ln^III^(NO_3_)_3_·*n*H_2_O to form insoluble precipitates when adding base, likely as the water is deprotonated and the resultant OH group bonds to more metals uncontrollably. So, whilst, the synthesis of high nuclearity clusters from teaH_3_ (or RdeaH_2_) implies deprotonation of the ligand to give bridging O^−^ groups, this must be done carefully. A different means of preventing the formation of the insoluble precipitates is the addition of bulky co-ligands, though this strategy can cap the formation of extended structures. This balancing act is clearly achievable, though may explain the lack of solvothermal success with these ligands, more energetic conditions being harder to tame.

## 6 Metallo-stars

### 6.1 Old: {Nb^V^_3_La^III^} and new: {Mn_3_^III^Ln^III^} and {Fe^III^_3_Ln^III^}

**Fig. 17:**
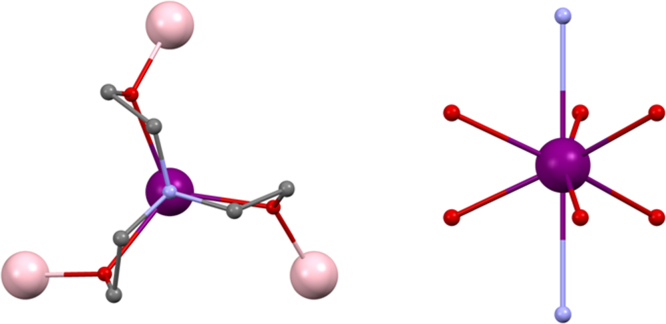
Left, a fragment of [Mn^III^_3_Ln^III^(tea)_3_(acac)_6_] **(24)** showing {Mn^III^_3_Ln^III^tea} only. Right, the ligand geometry around the central Ln^III^ ion. Key: Ln^III^, purple spheres; Mn^III^ pink; O, red; N, blue; C, grey; no H atoms are shown for clarity. See Ref. [52] for structural information.

An older and elegant gem is the first 4d–4f teaH_3_ compound, the metallo-star [Nb^V^_3_La^III^(tea)_2_(^i^PrO)_12_] **(23)** [51], although no magnetic properties were reported, as the metals are diamagnetic. The synthesis involves stirring [Nb^V^(^i^PrO)_5_] and “[H_3_La^III^(tea)_2_]”, in toluene. The H_3_ atoms of the latter are reported as the hydroxy-functionality of the tea^3−^ ligand, presumably three H atoms distributed between the six arms of two ligands for charge balancing. The final product has three outer Nb^V^ diamagnetic ions as the points of the star, each capped by four terminal (^i^PrO) groups. The two tea^3−^ ligands have the 4.2.2.2.1 mode, centred on La^III^, with one above and one below the plane. Whilst no analogous structures to **(23)** with paramagnetic 4f ions were reported, the first 3d–4f metallo-stars [52] are similar: [Mn^III^_3_Ln^III^(tea)_2_(acac)_6_][Mn^III^(acac)_3_] **(24)**, shown in Fig. 17, left, and [Fe^III^_3_Ln^III^(tea)_2_(acac)_6_] **(25)** (Ln^III^ = Gd^III^ or Dy^III^ in both cases). Synthesis was by refluxing [M^III^(acac)_3_], where M^III^ is Mn^III^ or Fe^III^, Ln^III^(NO_3_)_3_·*n*H_2_O, teaH_3_ and triethylamine in methanol, with crystals grown from a dichloromethane: hexane solution. These structures are identical save for the additional [Mn^III^(acac)_3_] unit in the former, and have the same tea^3−^ arrangement and mode as in **(23)**, visible as the polar ligands around the central ion, shown in Fig. 17, right; completing the octahedral environment of the 3d metals are two (acac)^−^ ligands, bonding terminally (1.1.1) to the outer metals. Thus, here, a balance between deprotonating the teaH_3_ and steric bulk has given a compact arrangement of metals with a large number of metals bonded to the amino-alcoholate. Despite the similar structures their magnetic properties are pleasingly distinct. For **(Gd-24)**, using the isotropic Heisenberg–Hamiltonian in Eq. (6), *J*_xy_, the interaction between Mn^III^ and Gd^III^, was weakly antiferromagnetic at −0.23 cm^−1^ and *J*_*yy*_, between Mn^III^ ions, a slightly stronger 0.56 cm^−1^, as fitted to *χT* data, which qualitatively agree with that for **(Dy-24)**, though other effects of Stark level depopulation with decreasing temperature may account for this behaviour in the latter.

(6) $$\hat{H}=-2\sum J({\hat{S}}_{x}{\hat{S}}_{y})$$

This means the Gd^III^ spins align opposite to that of the central ion, though this does not give an isolated ground state. The Fe^III^ compounds **(Gd-25)** and **(Dy-25)** show contrasting magnetic exchange; the former has *J*_xy_ = 0.73 cm^−1^ (between heterometals) and *J*_*yy*_ = −0.30 cm^−1^ (between like metals) giving an *S* = 11 ground state, *i.e.* where all spins are aligned. **(Dy-25)** was qualitatively similar. So far a.c. susceptibility studies are yet to be reported on these compounds, there being no other 3d–4f metallostars with which one could speculate upon the likely results, particularly the effect of the equatorial bonding on the SMM behaviour of the dysprosium(III) compounds.

## 7 Double cubanes

### 7.1 Christou's {Mn^III^_2_Mn^II^_2_Ln^III^_2_}

**Fig. 18:**
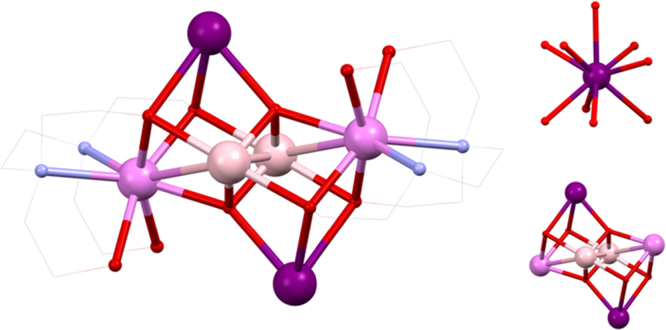
Left: skeletal view of [Mn^III^_2_Mn^II^_2_Ln^III^_2_O_2_(edteH_2_)_2_(O_2_C^*t*^Bu)_6_(NO_3_)_2_] **(26)**. Right, upper, geometry of Ln^III^ ion and core view, lower, of **(26)**: {Mn^III^_2_Mn_2_^II^Ln^III^_2_O_6_}. Key: Ln^III^, purple spheres; Mn^III^, palest pink; Mn^II^, pale pink/violet; O, red spheres; N, blue spheres (edteH_2_); C, grey wireframe; no H atoms, nitrates or pivalate groups are shown for clarity. See Ref. [53] for structural information.

Billed as the first 3d–4f double-cubane, [Mn^III^_2_Mn^II^_2_Ln^III^_2_O_2_(edteH_2_)_2_(O_2_C^*t*^Bu)_6_(NO_3_)_2_] **(26)** [53] (Ln^III^ = Gd^III^, Tb^III^, Dy^III^, Ho^III^ or Y^III^ and edteH_4_ = *N*,*N*,*N*′,*N*′-tetrakis(2-hydroxyethyl)ethylenediamine) is shown in skeletal view in Fig. 18, left, and incorporates a functionalised amine-diol giving four possible OH groups to bond and two N-donors. The structure is related to the incomplete double cubanes seen above, but maintains the missing metal vertices, so giving a hexa-metallic compound, the core of which is shown in Fig. 13, right and lower, We find the edteH_2_ groups link the Mn^II^ corner metals, BVS calculations being used to assign the oxidation states, to both inner Mn^III^ ions and one Ln^III^, with the mode 3.3.1 for each “half”, the first arm linking the hetero-metals, the second to only a Mn^II^, to which the N also bonds. For the complete ligand this mode is 4.3.3.1.1.1.1. Oxygen atoms connect one Ln^III^, Mn^II^ and both Mn^III^ ions with a tetrahedral arrangement. Carboxylate groups join each lanthanide to two Mn^III^ ions, each of these ligands bonding in 2.1.1 fashion between heterometals. A further carboxylate bonds terminally to each lanthanide in 1.1.1 fashion, with a terminal NO_3_, 1.1.1, completing the nine-coordinate geometry, given in Fig. 18, right and upper. The double-N bonding of edteH_2_ may encourage a preference of this ligand for Mn^II/III^ rather than Ln^III^ bonding.

**Fig. 19:**
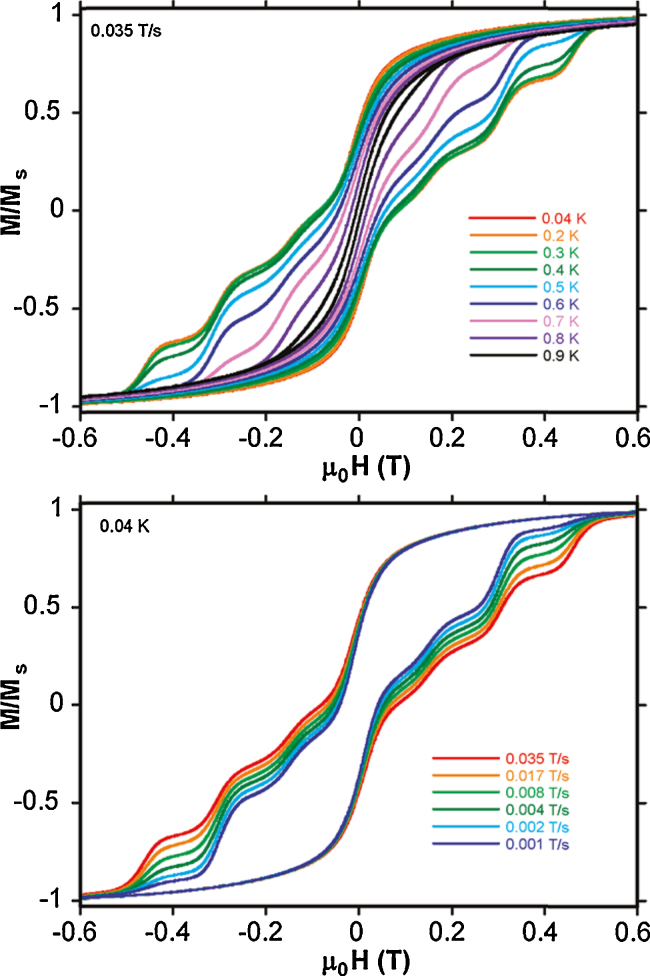
Hysteresis loops for **(Tb-26)**, for, upper panel, variable temperature, fixed sweep rate single crystal measurements between 0.04 and 0.9 K, and, lower panel, for fixed temperature, variable sweep rates of 0.001–0.035 T s^−1^.

Reprinted with permission from [53]. Copyright (2011) American Chemical Society.

Beautifully defined magnetic data were obtained from single-crystal studies of **(Tb-26)**, the only SMM of this series, reproduced in Fig. 19, upper panel, shows hysteresis loops opening below 0.9 K (0.035 T s^−1^) with a multi-step structure. This shows a significant zero-field QT relaxation. At 0.04 K the loop was sweep rate dependent, indicating QTM, as shown in the lower panel of Fig. 19. The Arrhenius plot shows the distinct thermal and quantum relaxation processes at 0.3 K, the former giving an energy barrier of 20 K, modest but significant. The choice of lanthanide(III) ion is important here, suggesting a tuning of the coordination environment. The Mn^III^ ions are believed to be unimportant in this regard, as they couple antiferromagnetically to give an *S* = 0 state.

### 7.2 Powell's {Fe^III^_4_Ln^III^_2_}

From the same ligand as above [Fe^III^_4_Ln^III^_2_O_2_(edteH)_2_(O_2_C^*t*^Bu)_6_(NO_3_)_2_]·*x*MeCN·*y*CH_2_Cl_2_·*z*C_6_H_5_OH **(27)** [54] (Ln^III^ = Gd^III^, Dy^III^ or Y^III^) has a similar formulation and structure to **(26)** though has a slightly more distorted metal core. Here, though, the edteH ligand bonds with a 4.2.2.2.0.1.1 mode where the OH ascribed is unbonded to a metal, and directs an H-bonded 1D network. Each Fe^III^ atom, which is bound to both the N-donor atoms in the poly-ol ligand, making up the outer metals of the {Fe^III^_4_} rhombus, is bonded to both the inner 3d metals and a lanthanide ion. Oxo ligands bridge between three metals of the rhombus directing triangular arrangements.

These compounds enabled a good understanding of the exchange interactions to be determined, by fitting to magnetic data. For **(Y-27)** using the Hamiltonian below (Eq. (7)) gave Fe-Fe interactions of *J*_outer_ = −4.17 cm^−1^ (antiferromagnetic) and *J*_inner_ = 1.1 cm^−1^ for *g* = 2.01. For **(Gd-27)** this was extended to the following Hamiltonian (Eq. (8)), giving *J*_outer_ = −4.4 cm^−1^, *J*_inner_ = 1.6 cm^−1^ and *J*_Fe outer-Gd_ = −0.12 cm^−1^, *J*_Fe inner-Gd_ = 0.24 cm^−1^.

(7) $$\hat{H}=-2J_{\text{outer}}({\hat{S}}_{1}{\hat{S}}_{2}+{\hat{S}}_{2}{\hat{S}}_{3}+{\hat{S}}_{3}{\hat{S}}_{4}+{\hat{S}}_{4}{\hat{S}}_{1})-2J_{\text{inner}}{\hat{S}}_{1}{\hat{S}}_{3}$$

(8) $$\hat{H}=-2J_{\text{outer}}({\hat{S}}_{1}{\hat{S}}_{2}+{\hat{S}}_{2}{\hat{S}}_{3}+{\hat{S}}_{3}{\hat{S}}_{4}+{\hat{S}}_{4}{\hat{S}}_{1})-2J_{\text{inner}}{\hat{S}}_{1}{\hat{S}}_{3}-2J_{\text{Fe}\;\text{inner}\text{-}\text{Gd}}({\hat{S}}_{1}{\hat{S}}_{5}+{\hat{S}}_{1}{\hat{S}}_{6}+{\hat{S}}_{3}{\hat{S}}_{5}+{\hat{S}}_{3}{\hat{S}}_{6})-2J_{\text{Fe}\;\text{outer-Gd}}({\hat{S}}_{2}{\hat{S}}_{5}+{\hat{S}}_{4}{\hat{S}}_{6})$$

These competing Fe^III^—Fe^III^ interactions were related to the bond length and bridging angle and matched with parameters from previous work.

For **(Dy-27)** an energy barrier, *U*_eff_, was 30.85 K in a d.c. field of 1200 Oe, though SMM behaviour was not seen for the other derivatives and so was assigned to the lanthanide ion. Mössbauer studies of these compounds revealed a trend of increasingly slow spin fluctuation from **(Y-27)** through **(Gd-27)** to **(Dy-27)**.

### 7.3 Thoughts on double cubanes

From these two similar compounds the extra donor atoms of the edteH_4_ ligand appear not to give extensive increases in the coordination, compared to tridentate RdeaH_2_ pro-ligands, or teaH_3_, despite its hexadentate nature. The propensity for the N atoms to anchor, here on the same atom, likely due to their proximity, may hinder the coordination and force the ligand to act as a capping group. Interesting is the change in protonation of this ligand to accommodate the charge balance, well demonstrating its flexibility, and also the change in bonding mode; although in the iron compound this is less protonated and the bonding is less extensive.

## 8 Double cubane variations

### 8.1 A former champion: {Mn^III^_4_Mn^IV^Ln^III^_4_}

[Mn^III^_4_Mn^IV^Ln^III^_4_O_6_(mdea)_2_(mdeaH)_2_(O_2_C^*t*^Bu)_6_(NO_3_)_4_(H_2_O)_2_]·2MeCN **(28)** [55] (Ln^III^ = Tb^III^, Dy^III^, Ho^III^ or Y^III^) has an intriguing core comprised of vertex-joined Ln^III^_2_Mn^III^_2_O_4_ cubanes similar to **(26)**, with a common Mn^III^, and two additional Mn^III^ ions bonded to two of the corner oxygen atoms.

A common Mn^IV^ links two {Ln^III^_2_Mn^IV^Mn^III^O_4_} cubanes with two axes defined by {Mn^III^_2_Mn^IV^} and {Ln^III^_4_Mn^IV^} planes. Outside of this are the “extra” two Mn^III^ ions, with aligned Jahn–Teller axes. O atoms linking these to the {Ln^III^_2_Mn^IV^} ions and bonding to the cubane metals {Ln^III^Mn^IV^Mn^III^}. The mdea^2−^ ligand bonds with the 4.3.2.1 mode joining a {Ln^III^_2_Mn^III^} unit in the cubane with one arm and connecting the N-bonded Ln^III^ with the non-cubane Mn^III^. For mdeaH^−^ the unprotonated arm centres on the extra Mn^III^, bridging this with a cubane Mn^III^, whilst the OH arm bridges with a Ln^III^ ion. NO_3_ and H_2_O groups bond to the lanthanide ions with pivalate groups bonding in 2.1.1 fashion between the cubane Ln^III^s and non-cubane Mn^III^, and hetero-metals in the cubane, *i.e.* Mn^III^ and Ln^III^s.

These were the first 3d–4f compounds using mdeaH_2_, with **(Dy-28)** showing the largest *U*_eff_ at the time for any 3d–4f compound, this being *ca*. 40 K and significantly less for the other variants, particularly **(Y-28)** (*ca*. 20 K). Micro-squid single crystal measurements revealed QTM, characterised by stepped hysteresis loops at 1.9 K (0.002 T s^−1^ sweep rate). Though there are two distinct lanthanide(III) geometries, one a tricapped triangular prism, the other a distorted square antiprism, there appears only one *χ*″ maximum. This may suggest only one ion is playing a role in the slow relaxation, though this must be in concert with the Mn^III^ ions.

### 8.2 Thoughts on double cubane variations

This familiar pattern of the dysprosium compound showing the largest *U*_eff_ in a series is likely due to Dy^III^'s status as a Kramers ion (with an odd number of electrons), whereby the ground state is always bi-stable in any crystal field environment, though not necessary optimised to separate the high magnitude *m*_*J*_ states from others. This is not true for Tb^III^ and Ho^III^, where symmetrical ligand environments are required, deviations impinging on the relaxation mechanism. In that sense it is “easier” to make a Dy^III^-based SMM, especially when the ligand geometry cannot be controlled as is the case with such flexible ligands. There is also a broader point about all of the compounds measured and analysed in this review. It is debatable, indeed unlikely, that any researchers have achieved the necessary degree of “control” over the ligand geometry that is the key for SMM optimisation; these ligands are amongst the most flexible families in the literature, along with phosphonic acids, perhaps, so this control is extremely difficult and probably impossible, in this context. The search for high performance SMMs, then, with these ligands, involves a good degree of luck. Lifting this somewhat gloomy outlook, as we move to the second section of this review, is the fact that there are very few ligands that can achieve reliable metal-topology control in molecular magnetism as a whole; there is still a certain alchemy in the synthesis of most SMMs and a hit-and-hope approach, we believe.

## 9 Lanthanides and copper

### 9.1 Le Corbusier's choice?: [Cu^II^_5_Ln^III^_4_O_2_(teaH)_4_(OMe)_4_(O_2_C^*t*^Bu)_2_(NO_3_)_4_]·2MeOH·2Et_2_O

The search for compounds with high-spin and low anisotropy as magnetic refrigerants led to several investigations with Cu^II^ (*S* = 1/2) and Gd^III^ (*S* = 7/2, ^8^S_7/2_), the most successful of these being [Cu^II^_5_Ln^III^_4_O_2_(teaH)_4_ (OMe)_4_(O_2_C^*t*^Bu)_2_(NO_3_)_4_]·2MeOH·2Et_2_O **(29)** [56], [57] (Ln^III^ = Gd^III^, Tb^III^, Dy^III^ or Ho^III^). This was made by stirring Cu^II^(NO_3_)_2_·3H_2_O, Ln^III^(NO_3_)_3_·*n*H_2_O, pivalic acid, triethylamine and teaH_3_ in acetonitrile and layering the resulting solution with ether and gives an unusual structure of a {Cu^II^_5_} “bow-tie”, with one Ln^III^ above and one below each of the two “nodes.” The single type of teaH^2−^ has two bonding modes, mode one being 3.2.2.0.1 (as for **(6)**) and therefore bonding between a “terminal” copper and lanthanide ion with one O-arm and between the same copper and separate lanthanide with a second O-arm; the OH arm is free and N bonds to this same terminal copper; mode two bonds to more metals as 4.2.2.2.1. Overall this bonds between two terminal coppers and two lanthanides above and below the same node: the first arm thus bonds between the copper ions, the second arm to a copper and lanthanide and the third between this same copper, to which N bonds, and different lanthanides.

One of the sensible rationales for targeting such Gd^III^Cu^II^ compounds for their MCE properties is the expected ferromagnetic exchange between these heterometals; *χT* increases at low temperatures, though this could be assigned to several other interactions, *vide infra*. Indeed, −Δ*S*_*M*_ = 31 J kg^−1^ K^−1^ (Δ*H* = 9 *T*, 3 K) for **(Gd-29)** going alongside a large Δ*T*_AD_, one of the largest for any molecule.

The SMM behaviour of the anisotropic variants revealed small energy barriers using a non-conventional (non-Arrhenius) method, giving *U*_eff_ as 12, 7 and 10 K for **(Tb-29)**, **(Dy-29)** and **(Ho-29)** compounds, respectively.

Amusingly, for **(Gd-29)** a fitting of magnetic data using the Hamiltonian in Eq. (9) revealed that the Cu^II^—Gd^III^ was actually antiferromagnetic, though competing Cu^II^—Cu^II^ interactions and ferromagnetic Gd^III^—Gd^III^ coupling were also observed, so the explanation is not straightforward.

(9) $$\hat{H}=-\sum J({\hat{S}}_{x}{\hat{S}}_{y})$$

**Fig. 20:**
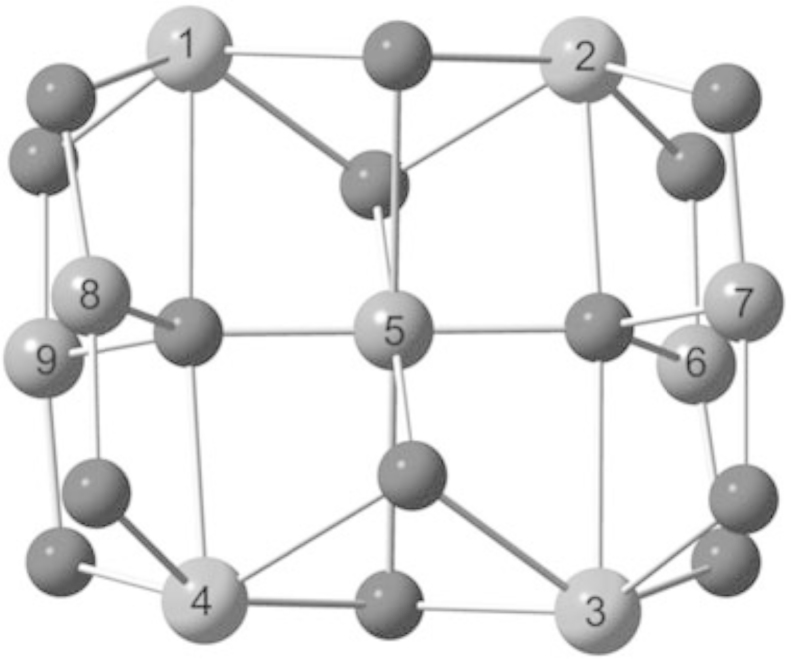
Numbering scheme for **(29)**, where 1–4 are Ln^III^ ions and 5–9 are Cu^II^ ions.

Reproduced with permission from [57]. Copyright 2011, Wiley-VCH Verlag GmbH & Co. KGaA.

Further study with **(Dy-29)** revealed dominant weak ferromagnetic coupling though this is composed of several different interactions, namely a rather complicated coupling scheme with the Hamiltonian given below (Eq. (10)) with each spin operator given using the numbering system shown in Fig. 20.

(10) $$H=-J_{1}({\hat{S}}_{1}{\hat{S}}_{5}+{\hat{S}}_{1}{\hat{S}}_{8}+{\hat{S}}_{1}{\hat{S}}_{9}+{\hat{S}}_{2}{\hat{S}}_{5}+{\hat{S}}_{2}{\hat{S}}_{6}+{\hat{S}}_{2}{\hat{S}}_{7}+{\hat{S}}_{3}{\hat{S}}_{5}+{\hat{S}}_{3}{\hat{S}}_{6}+{\hat{S}}_{3}{\hat{S}}_{7}+{\hat{S}}_{4}{\hat{S}}_{5}+{\hat{S}}_{4}{\hat{S}}_{8}+{\hat{S}}_{4}{\hat{S}}_{9})-J_{2}({\hat{S}}_{5}{\hat{S}}_{6}+{\hat{S}}_{5}{\hat{S}}_{7}+{\hat{S}}_{5}{\hat{S}}_{8}+{\hat{S}}_{5}{\hat{S}}_{9})-J_{3}({\hat{S}}_{6}{\hat{S}}_{7}+{\hat{S}}_{8}{\hat{S}}_{9})$$

In contrast to this, the expected ferromagnetic Cu^II^—Dy^III^ interaction was present, *J* = +1.0 cm^−1^, with competing interactions between inequivalent pairs of Cu^II^ ions of +1.3 and −4.6 cm^−1^, though the Dy^III^—Dy^III^ interaction was negligible. Despite the differences these compounds vindicate Ln^III^—Cu^II^ teaH_3_ compounds both as an approach to molecular refrigerants, using Gd^III^, and as SMMs, when anisotropic lanthanides are included.

## 10 Why so Cerious?

### 10.1 ^*t*^BudeaH_2_ and {Ce^III/IV^Mn^III^}

[Ce^IV^_6_Ce^III^_2_Mn^III^_2_O_8_(^*t*^Budea)_2_(O_2_C^*t*^Bu)_12_(NO_3_)_2_(O_2_CCH_3_)_2_]·4CH_2_Cl_2_ **(30)**, [Ce^IV^_8_Ce^III^Mn^III^O_6_(OH)_3_(^*n*^Budea)_4_(O_2_C^*t*^Bu)_9.5_(NO_3_)_3.5_(O_2_CCH_3_)_2_]·1.5MeCN **(31)** and [Ce^IV^_4_Mn^IV^_2_O_4_(^*n*^Budea)_2_(O_2_C^*t*^Bu)_10_(NO_3_)_2_]·4MeCN **(32)** are amine-diol compounds amongst several {Ce^III/IV^Mn^III^} structures prepared [58], these being subject to single crystal XRD and other characterisation techniques. Similar preparations were used for each of these, namely stirring Mn^II^(O_2_CCH_3_)_2_·4H_2_O Ce^III^(NO_3_)_3_·*n*H_2_O, pivalic acid and the appropriate RdeaH_2_, in varying ratios. **(32)** also requires the addition of [NH_4_]_2_[Ce^IV^(NO_3_)_6_]. As these have no magnetic studies attached they will only be described briefly:

The first of these, **(30)**, is based around a {Ce^IV^_6_} octahedron, where N-bonded Mn^III^ ions are linked to an equatorial Ce^IV^ by a ^*t*^Budea^2−^ ligand, which bonds this same 3d metal to an equatorial Ce^IV^, hence the 3.2.2.1 mode. The Mn^III^—N bond defines the Jahn–Teller axis. The second compound **(31)** also displays the 3.2.2.1 mode, the amino-alcoholate centred on both a Mn^III^, bridging to two lanthanides and the remaining four ligands centred on a lanthanide, bridging to two further lanthanides. The last, **(32)**, also shows the 3.2.2.1 mode, centred on a Mn^IV^, through which a N—Mn^IV^—O Jahn–Teller axis is defined.

### 10.2 {mdeaH_2_ and Ce^III^}

[NaCe^III^_10_O_7_(mdea)_5_(OH)(ib)_14_(O_2_CH)] **(33)** [59] (ib = isobutyrate) was characterised structurally only, having been synthesised from the starting material [Ce^III^_2_(ib)_6_(H_2_O)_3_]_*n*_, mdeaH_2_, sodium isobutyrate and acetonitrile using the uncommon reaction method of ultra-sonication. This can be thought of as having a cerium(III) hexanuclear core capped by a {Ce^III^_3_Na} tetrahedron. Briefly, there are three bonding modes of mdea^2−^, three of the five ligands displaying the 3.2.2.1 mode to a trio of cerium ions, and two with the 4.3.2.1 mode, though one of these bonds a sodium ion onto the higher denticity arm.

An exciting prospect for all these compounds from the 4f perspective, would be the inclusion of more magnetically “interesting” ions, such as Dy^III^ or Gd^III^, though these would require changes in ligands to balance charges where Ce^IV^ is involved.

## 11 Tea for one

### 11.1 Monomeric teaH_3_ compounds

A number of monometallic lanthanide(III) compounds with various forms of teaH_3_ ligand have been recorded over the past 25 years or so. Several of these contain fully protonated teaH_3_ bonding to a single metal, the charge being balanced by various anions, for example [Ln^III^(teaH_3_)_2_(CF_3_SO_4_)_3_]·3THF **(34)** [60] (Ln^III^ = Pr^III^, Yb^III^ or Lu^III^, and THF is tetrahydrofuran) was synthesised by Hahn and Mohr by partial replacement of a labile triflate with teaH_3_ in THF. These have a nine co-ordinate capped square-antiprismatic geometry, due to the two amino-alcohol ligands bonding with the 1.1.1.1.1 mode and a single THF. This is related to [La^III^(teaH_3_)_2_(NO_3_)_2_]·2NO_3_ **(35)** [61], investigated by Fowkes and Harrison much more recently, where one NO_3_ bonds to the 4f ion with two O-atoms, making a ten co-ordinate central ion. These arrange into a one-dimensional supra-molecular structure by H-bonding through the OH of a teaH_3_ and the O of an unbound NO_3_ anion separating the lanthanide units.

Two Ln^II^ monometallic compounds, [Ln^II^(teaH_3_)_2_]·2ClO_4_ **(36)** [62] Ln^II^ = Eu^II^ or Yb^II^ were prepared by electrochemical reduction of the Ln^III^ analogues. The 1.1.1.1.1 bonding mode of teaH_3_ is present in each.

Burin and Bochkarev prepared insoluble [Ln^III^(tea)] **(37)** [63] (Ln^III^ = Y^III^, Nd^III^ or Er^III^; and Eu^III^), likely a polymeric compound, by a variety of methods, and also [Y^III^(teaH_2_)_3_] **(37b)** with the 1.1.0.0.0 bonding mode and [(Me_3_Si)_2_NY^III^(OC_2_H_4_)_2_NC_2_H_4_OY^III^][N(SiMe_3_)_2_]_2_·(THF) **(37c)**. The latter has an unusual bonding mode where the tea^3−^ ligand bonds to metals that are otherwise unsupported, and the N is not bonded to any metal, hence the 2.1.1.1.0 mode. The monometallic form of these is likely due to the protonated nature of the triethanolamine ligands, supporting the “common-sense” view that a negatively charged ligand is more likely to seek out more metals and give larger metal cages.

## 12 Purity

### 12.1 Open wide: {Ln^III^_6_}

**Fig. 21:**
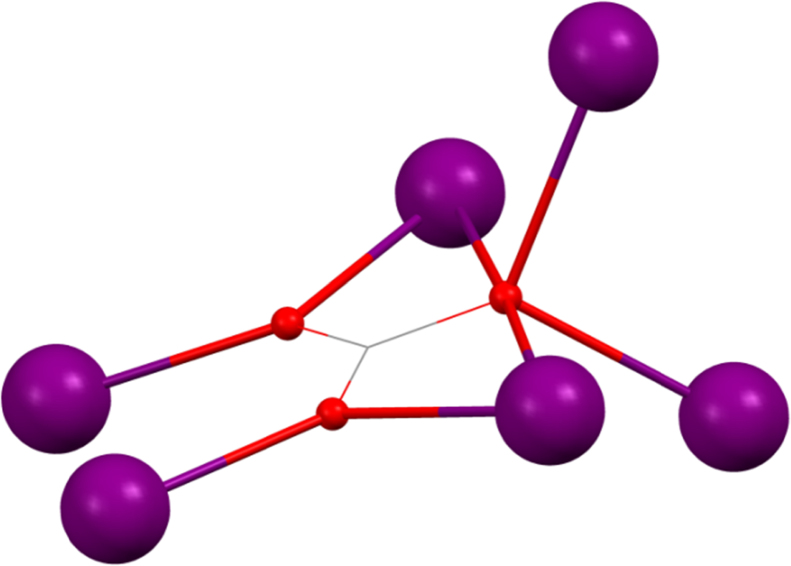
Core of [Ln^III^_6_(teaH)_2_(teaH_2_)_2_(CO_3_)(NO_3_)_2_(chp)_8_(H_2_O)] **(38)** showing only the six Ln^III^ ions and (CO_3_)^2−^ ligand. Key: Ln^III^, purple spheres; O, red spheres. See Ref. [64] for structural information.

Quantitatively similar behaviour to **(Gd-18)** and **(Dy-18)** was seen in a second {Ln^III^_6_} compound. From a similar synthesis to that of the first, *vide supra*, but with the addition of chpH (6-chloro-2-hydroxy-pyridine), a [Ln^III^_6_(teaH)_2_(teaH_2_)_2_(CO_3_)(NO_3_)_2_(chp)_8_(H_2_O)](NO_3_)·4.5MeOH·1.5H_2_O **(38)** [64] (Ln^III^ = Gd^III^, Tb^III^ or Dy^III^) cage was prepared. The structure can be described as having four planar metal ions, each with distorted square-antiprismatic geometries, with one above and one below where a fifth would be to form an open “mouth”. Then looking into this mouth, chp ligands bridge between the planar ions, with N and OH atoms, the rear two metals being bridged by two ligands in this way. Two chp ligands cap each of the two rearward metals by the OH group. The mouth ions are bonded to one NO_3_ group each. teaH^2−^ has the 3.2.2.1.1 mode, bridging between one mouth ion and two planar ions, a common enough motif where three ions are linked. The teaH_2_^−^ ligand has the 3.3.1.1.1 mode, where O-arms bond between the two mouth and an outer lanthanides, with the co-ordinating atoms bonding only to the planar, outer ion. Finally a carbonate, seemingly derived from CO_2_ in the atmosphere supports the structure, bonding to all metals with a 6.2.2.2 mode, seen in Fig. 21, two further interactions of one O-atom being around 2.8 Å, which were not denoted as bonds. Magnetic measurements show decreasing *χT* with decreasing temperature, for **(Gd-38)** this being likely due to antiferromagnetic interactions. For **(Tb-38)** and **(Dy-38)** this is probably due to the anisotropy of these ions, a suggestion backed up by magnetisation curves which are non-overlapping. The very small energy barriers, *U*_eff_, for these two compounds were 4.8 and 3.8 K respectively under zero field, though no visible peaks in *χ*″ *versus T* (*ν*) were found. Further work will give a deeper understanding of the exchange and anisotropy parameters in these compounds.

### 12.2 Homo-metallic dysprosium compounds

As this article was in press two new and exciting homo-metallic dysprosium compounds were reported [65]: [Dy^III^_3_(OH)(teaH_2_)_3_(paa)_3_]Cl_2_·MeCN·4H_2_O **(39)**, where paaH is *N*-(2-pyridyl)-acetoacetamide, and [Dy^III^_8_)OH)_6_(teaH)_6_(teaH_2_)_2_(teaH_3_)_2_](O_3_SCF_3_)_4_·0.5MeOH·2H_2_O **(40)**. The first has a triangular core where the teaH_2_ ligands bond with the 2.2.1.1.1 mode, centred on one Dy^III^ each. No SMM behaviour was observed, and a finite (*i.e.* non-zero) magnetisation occurred at the low temperatures, so this does not have a toroidal spin arrangement, *vide supra*. For **(40)**, the synthesis is related to that of the {Ln^III^_6_} wheels above by a change in salt used. The three types of amine-polyol result support a structure of three fused butterflies. Several bonding modes are exhibited by the three different kinds of amino-polyol:teaH^2−^ shows the 2.2.1.1.1 mode; teaH_2_^−^ shows the 3.3.1.1.1 and 2.2.1.1.1 modes; the teaH_3_ ligand has the 1.1.1.1.1 mode. This compound shows possible SMM behaviour below 8 K, though no frequency dependent maxima were observed in the a.c. susceptibility experiment.

## 13 More cages

### 13.1 {Fe^III^_7_Ln^III^_4_}

More work with reactions of Fe^III^ triangles gave [Fe^III^_7_Ln^III^_4_O_4_(OH)_3_(tea)_2_(teaH)_3_(O_2_C^*t*^Bu)_7_(NO_3_)_2_(H_2_O)_2_](NO_3_)·3MeCN **(41)** [66] (Ln^III^ = Dy^III^ or Y^III^) when [Fe^III^_3_O(O_2_C^*t*^Bu)_6_(H_2_O)_3_](O_2_C^*t*^Bu), Fe^III^(NO_3_)_3_·9H_2_O, Ln^III^(NO_3_)_3_·*n*H_2_O and teaH_3_ were refluxed in acetonitrile and the organic fraction separated for further heating, crystals growing from this solution. This structure consists of what would be a butterfly {Fe^III^_4_} core, though one inner site is split between two Fe^III^ ions. This {Fe^III^_5_O_7_} unit is capped by two further Fe^III^ ions on two vertices. The inner Fe^III^ ion is also a common vertex of two {Fe^III^_2_Dy^III^_2_} cubanes, giving a rather complex core.

There are distinct tea^3−^ ligands here which bond with the 3.2.2.1.1 mode, this being Fe—N bonded and linked to two lanthanide ions. The second has the 4.2.2.2.1 mode linking pairs of metals with a common iron. Furthermore there are three types of teaH^2−^: these have the modes 4.3.2.1.1, linking a central N-bonded dysprosium(III) to two heterometals with one arm and another iron(III) with a second arm. Unusually the third, protonated, arm is bonding the central (lanthanide) ion; the 3.2.2.0.1 mode, which is common when one arm is protonated, linking an iron(III) to two adjacent lanthanides; and a second 3.2.2.0.1 mode where the iron is bridged to heterometals. (O_2_C^*t*^Bu) groups show diversity in their bonding, too, with two bridging pairs of lanthanides, three bridging pairs of irons, and capping groups with the modes 1.1.1 and 1.1.0 to lanthanide ions. Nitrates cap 1.1.0 to iron ions and there is also one water on each of one iron(III) and one dysprosium(III). Magnetic data were ambiguous regarding the interactions within this compound, with an explanation for the increase in *χT* at low temperatures being sought with ^57^Fe Mössbauer spectroscopy, which showed an antiferromagnetic exchange between these 3d ions and a ferromagnetic exchange between the Dy^III^ ions.

### 13.2 {Fe^III^_7_Dy^III^_3_}

[Fe^III^_7_Dy^III^_3_O_2_(OH)_2_(mdea)_7_(O_2_CC_6_H_5_)_4_(N_3_)_6_]·2H_2_O·7CH_3_OH **(42)** [67], a decametallic cage, was formed from methanolic solutions of Fe^III^Cl_3_, Dy^III^Cl_3_·6H_2_O, NaN_3_, benzoic acid and mdeaH_3_, growing crystals from the refluxed mixture. Each ion is unique in the crystal structure, which is based around a {Fe^III^_3_Dy^III^_3_} core, where O bridges {Dy^III^_2_Fe^III^_2_} units. OH groups link the outer {Dy^III^_2_} part of this core to an outer Fe^III^ which is linked in turn by a mdea^2−^ ligand to a different Fe^III^ and one of the core Dy^III^ ions. All Fe^III^ ions have an mdea^2−^ ligand centred on them, all with the 3.2.2.1 mode. Three mdea^2−^ groups link {Fe^III^_2_Dy^III^} units, and four link together {Fe^III^Dy^III^_2_} parts of the structure, which also incorporates benzoate groups bridging hetero-metals. Six N_3_ ligands bond terminally to all 3d metals with the exception of one “core” iron. The energy barrier, *U*_eff_, for this SMM was *ca*. 33 K, with no quantum tunnelling observed from the single crystal experiment in an applied field, though this effect was present when *H*_d.c._ = 0, and there was hysteresis below 2.0 K (0.035 T s^−1^). Mössbauer studies were also able to establish a barrier to relaxation below 35 K. Rationalising the rather low barrier value one may point to the slightly distorted square-antiprismatic geometry around the lanthanide(III) ion, with a range of bond lengths.

### 13.3 {Mn^III^_9_Dy^III^_8_}

The hepta-decametallic cage [Mn^III^_9_Dy^III^_8_O_8_(OH)_8_(tea)_2_(teaH)_2_(teaH_2_)_4_(O_2_CCH_3_)_4_(NO_3_)_2_(H_2_O)_4_](NO_3_)_7_·8H_2_O **(43)** [68], which shows signs of SMM behaviour at low temperatures is synthesised from Mn^II^(NO_3_)_2_·4H_2_O, Dy^III^(NO_3_)_3_·*n*H_2_O, sodium acetate, triethylamine and teaH_3_, which were stirred in methanol:acetonitrile with crystals obtained from the ether layered solution. That slight variations in synthesis can lead to large differences in topology should now be apparent, which we could rationalise here by the sterically small ligands unable to stabilise a relatively low nuclearity structure by encapsulation.

One way to represent the structure is to see it as based around an almost planar {Mn^III^_5_Ln^III^_2_} disc. Linked to this and offset above and below is a {Ln^III^_2_Mn^III^_2_} triangular based pyramid with outlying Ln^III^ atoms almost in the disc plane on either side giving the seventeen metal core, held together by O and OH ligands, pivalates and teaH_3_ derivatives. NO_3_ and H_2_O groups are only terminal here. Depending on how deprotonated the teaH_3_ ligand is, three different bonding modes are seen, namely the highly coordinating 5.3.2.2.1 for tea^3−^, which, joins a basal “pyramidal” Dy^III^ with a planar and basal Mn^III^; to a planar Dy^III^; and to an apical Dy^III^; 3.2.2.1.1 for teaH^2−^, these joining a “disc” Dy^III^ to adjacent “disc” Mn^III^ ions; and 2.2.1.1.1 for teaH_2_^−^, these linking an outlying Dy^III^ to a disc Mn^III^, repeated for the apical Dy^III^ of the pyramid unit, all having N—Dy^III^ bonding.

*χT*(*T*) is constant down to 25 K before increasing significantly from a room temperature value of *ca*. 132 cm^3^ mol^−1^ K (in line with that expected for uncoupled ions) to a 3 K value of *ca*. 211 cm^3^ mol^−1^ K. This would imply ferromagnetic interactions between ions, though the decrease in *χT*(*T*) below this temperature also suggests some antiferromagnetic interactions (or a depopulation of Stark sublevels) are present. Quantifying the SMM behaviour of **(43)** was hindered by the lack of maxima in *χ*″, even at low temperatures in the a.c. susceptibility experiment.

### 13.4 Organo-metallics: {Mn^III^_4_Nd^III^_4_}

Taking a larger 3d starting material, here [Mn^III^_2_Mn^II^_4_O_2_(O_2_C^*t*^Bu)_10_(4-Me-py)_2.5_(HO_2_C^*t*^Bu)_1.5_] **(44)** [69] and reacting this with ^*n*^BudeaH_3_, Nd^III^(NO_3_)_3_·*n*H_2_O and ferrocene (Fe^II^) dicarboxylic acid (fcdcH_2_) in acetonitrile gave the [Mn^III^_4_Nd^III^_4_(OH)_4_(^*n*^Budea)_4_(fcdc)_2_(O_2_C^*t*^Bu)_8_]·H_2_O square-wheel, which is an almost planar arrangement of a 4f square inside a 3d one. Pivalate groups frame the whole by bridging adjacent pairs of metals in 2.1.1 fashion and ^*n*^Budea^2−^ centres on the corner Mn^III^ ions and bridges between this and the neighbouring lanthanides in a 3.2.2.1 manner. OH groups centre between two inner Nd^III^ ions and a corner Mn^III^. One fcdc^2−^ sits above and another below the metal core, only bonding to Nd^III^ ions *via* the carboxylate groups. Interestingly this gives three different coordination numbers of 8, 8, 9 and 10 for each Nd^III^. The *χT*(*T*) behaviour at low temperature, signifies a rapid low temperature decrease that suggested overall antiferromagnetic interactions, though cautioned by the anisotropy of the Nd^III^ ion. Further investigations will seek SMM behaviour, perhaps with Dy^III^ and Tb^III^ ions as the Mn^III^ anisotropy is expected to be very small as their Jahn–Teller anisotropy axes will cancel out, from geometrical considerations. Nevertheless, the combination of conventional molecular magnetism synthesis with an organo-metallic staple (fcdcH_2_), though this basically acts as a “conventional” dicarboxylate, shows promise. Whether the Fe^II^ ion can be replaced by a more anisotropic ion is a further question to ponder. Furthermore, the large differences in geometry at the metals would make a very interesting test for resolution of any differences in relaxation at each individual site, already seen for at least two different coordination environments in Dy^III^ compounds.

### 13.5 Similar but different: {Fe^III^_5_Ln^III^_8_} and {Mn^III^_5_Ln^III^_8_}

**Fig. 22:**
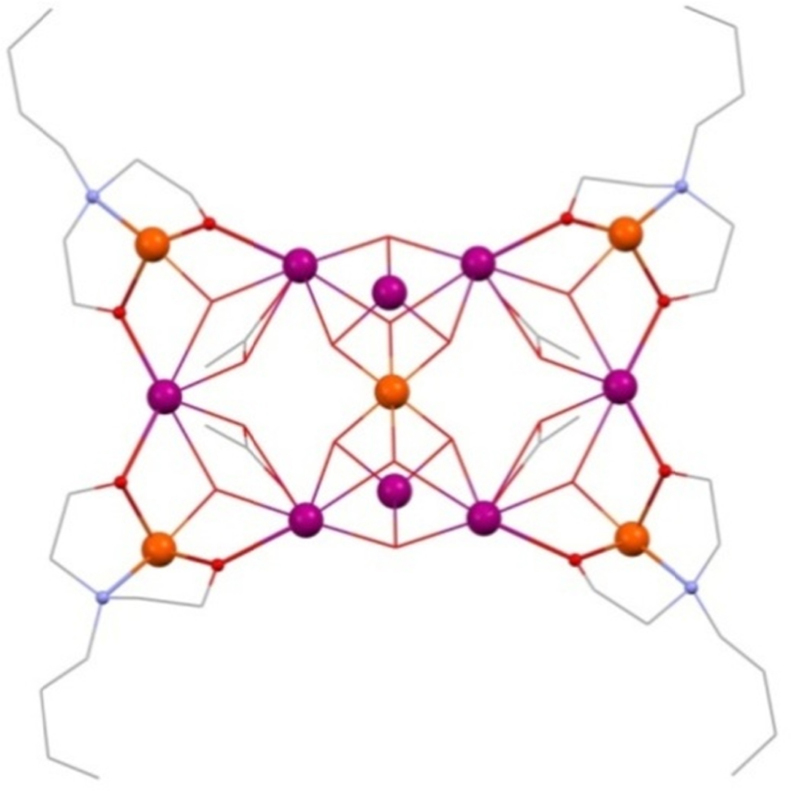
[Fe^III^_5_Ln^III^_8_(OH)_12_(^*n*^Budea)_4_(O_2_C^*t*^Bu)_12_(NO_3_)_4_(O_2_CCH_3_)_4_] **(46)**. Key: Ln^III^, purple spheres; Fe^III^, orange; O, red spheres (^*n*^BudeaH_2_) and wireframe (carboxylates); N, blue spheres (^*n*^BudeaH_2_); C, grey wireframe; no H atoms, NO_3_ or pivalate groups are shown. See Ref. [71] for structural information.

A series of analogous complexes, using Fe^III^ and Mn^III^ is the tridecametallic [M^III^_5_Ln^III^_8_(OH)_12_(Rdea)_4_(O_2_C^*t*^Bu)_12_(NO_3_)_4_(O_2_CCH_3_)_4_]^−^, where M^III^ is Mn^III^ **(45)** [70] (Ln^III^ is Pr^III^, Nd^III^, Sm^III^, Gd^III^ or Tb^III^, R is ^*t*^Bu) or Fe^III^ **(46)** [71] (Ln^III^ is Pr^III^, Nd^III^ or Gd^III^, R is ^*n*^Bu) (Fig. 22). Despite the similarities in the structure, the syntheses are different, though they could be viewed as having the same parts in a different order. **(45)** requires addition of the ^*t*^BudeaH_2_ ligand in acetonitrile to Mn^II^(OAc)_2_·4H_2_O, Ln^III^(NO_3_)_3_·*n*H_2_O and pivalic acid in acetonitrile and stirring at room temperature. Crystals were grown from the filtered mixture. For **(46)**, the source of Fe^III^ and pivalate was [Fe^III^_3_O(O_2_C^*t*^Bu)_6_(H_2_O)_3_](O_2_C^*t*^Bu), which was stirred with NaOAc·3H_2_O, ^*n*^BudeaH_2_ and Ln^III^(NO_3_)_3_·*n*H_2_O in acetonitrile, giving crystals directly.

The tri-decametallic topology common to both is based around a core of vertex sharing triangular-based pyramids, where only their common vertex is a M^III^, other ions being lanthanides. This is bonded to by two of the “basal” lanthanides making up triangles of {Ln^III^_2_M^III^}. These are themselves linked by a common lanthanide vertex. The four triangular faces of the pyramid are capped by OH groups, which also centre the aggregated triangles. Pivalate groups “frame” the structure, all bonding 2.1.1. **(46)** was in fact the first 3d–4f compound incorporating ^*n*^BudeaH_2_, in 2007. Viewing this compound such that it appears as a “rectangle”, four Rdea^2−^ ligands centre on each of the corner 3d transition metals, bonding with the mode 3.2.2.1, as previously seen (see Scheme 1) to two lanthanides. Nitrates adopt a familiar role in capping 1.1.1.0 onto the innermost pyramid-type lanthanides.

Comparing the magnetic properties of these two series we find the following: The Mn^III^ compounds were synthesised in the hope of finding SMM behaviour lacking in any Fe^III^ derivative. Unfortunately this was unsuccessful and ascribed to the amusingly named “magnetic death zone”, a region whereby the Mn^III^—O—Ln^III^ and Ln^III^—O—Ln^III^ bond angles and lengths lead to extremely weak interactions between spins, behaviour which was extrapolated from the Gd derivative of the Fe^III^ and Mn^III^ compounds, where *χT* increases only at low temperatures, signifying overall weak ferromagnetic coupling. In neither case, though, were any fits of the magnetic data to a spin coupling model obtained. Also, this does not explain why single-ion effects were not apparent, as individual lanthanide(III) ions can produce enormous energy barriers, though the dysprosium(III) compound was not prepared which is the most likely candidate.

### 13.6 Mixing it up

Recently demonstrating that combinations of conceptually similar ligands can be profitable is **(47)** [72], [Mn^II^_2_Mn^III^_2_Ln^III^_2_(O_2_C^*t*^Bu)_8_(thme)_2_(teaH_2_)_2_], where thmeH_3_ is tris(hydroxymethyl)ethane and Ln^III^ is Pr^III^—Dy^III^, excepting Pm^III^). There are two separate tripodal alcohol ligands employed here to give a structure made up of a bicapped Mn^II^_2_Ln^III^_2_ defect cubane. Although neither of **(Tb-47)** or **(Dy-47)** show confirmed SMM behaviour, the presence of frequency-dependent signals in the a.c. susceptibility suggest these may behave as such, at lower temperatures. The bonding mode of teaH_2_^−^ is seen elsewhere, being 2.2.1.1.1 and centred on a Ln^III^ ion.

### 13.7 Thoughts on cages

The myriad cages here demonstrate how many different topologies and shapes of cages could be waiting to be discovered. Particularly interesting is the use of organometallic ligands, which could open up interesting new avenues to explore.

## 14 Conclusions

We hope to have shown that the ligands teaH_3_ and RdeaH_2_ are extremely versatile, flexible and so are useful tools in the synthetic chemistry of hard metal ions, leading to a wide variety of structurally diverse and topologically interesting molecules. These have shown how a single ligand can bond up to seven ions and down to a single one. This could lead to promising magnetocaloric materials, where the ligand to metal ratio is crucial in determining the usefulness of such compounds. One problem here may be a lack of control of the magnetic exchange, which is not just a limitation of polyaminoalcoholates, though. When one considers SMMs, further downsides of these ligands become apparent, for instance, manipulating the exchange between spins, or the alignment of Mn^III^ anisotropy axes, as factors such as the length of the pendant arms, variable donor-atom-metal interactions and variations in denticity make targeted syntheses conceptually difficult.

We have described some indications as to why certain compounds show such slow relaxation though more work is clearly required on several more compounds to understand this, through factors of exchange interactions, molecular symmetry and coordination environment, which are again hard to target reliably in most areas of molecular magnetism, and especially when there is also the complication of such flexible ligands.

These topological problems are challenges that we feel will not be met by these ligands. Nevertheless several important results have been made incorporating them, such as high performance refrigerants and energy barriers, *U*_eff_, in SMMs, up to *ca.* 170 K **(Dy-9)**. There are also problems of assessing why 3d–4f or 4f SMMs behave in the way they do. Whilst square anti-prismatic geometries are generally favoured in Dy^III^ and Tb^III^ SMMs, the discovery of one does not lead to the other. This is because there are far too many parameters hidden behind such a facile description, which describe the crystal field and simplistic descriptions often hide quite substantial distortions from the “real” geometry. These ligands also have none of the redox chemistry that promises so much in the improvement of hysteresis temperatures in lanthanide(III) SMMs [29]. Whilst we could go on, we should perhaps merely say that these ligands are fun for chemists, who probably just want to make *something* with a new structure, but less so for physicists who may *know* what they want.
